# Synthesis, Crystal Structure, and Photoluminescent Properties of 3,3′,4,4′-Tetraethyl-5,5′-divinyl-2,2′-bipyrrole Derivatives

**DOI:** 10.3390/molecules22111816

**Published:** 2017-10-26

**Authors:** Toru Okawara, Reo Kawano, Hiroya Morita, Alan Finkelstein, Renjiro Toyofuku, Kanako Matsumoto, Kenji Takehara, Toshihiko Nagamura, Seiji Iwasa, Sanjai Kumar

**Affiliations:** 1 Department of Creative Engineering, National Institute of Technology, Kitakyushu College, 5-20-1 Shi-i, Kokuraminami-ku, Kitakyushu 802-0985, Japan; takehara@kct.ac.jp (K.T.); nagamura@kct.ac.jp (T.N.); 2 Advanced School of Creative Engineering, National Institute of Technology, Kitakyushu College, 5-20-1 Shi-i, Kokuraminami-ku, Kitakyushu 802-0985, Japan; ad1506rk@apps.kct.ac.jp (R.K.); ad1634hm@apps.kct.ac.jp (H.M.); 3 Department of Chemistry and Biochemistry, Queens College, Queens, NY 11367, USA; afinkelstein65@gmail.com (A.F.); Sanjai.Kumar@qc.cuny.edu (S.K.); 4 Department of Materials Science and Chemical Engineering, National Institute of Technology, Kitakyushu College, 5-20-1 Shi-i, Kokuraminami-ku, Kitakyushu 802-0985, Japan; c51228rt@apps.kct.ac.jp (R.T.); c51237km@apps.kct.ac.jp (K.M.); 5 Department of Environmental and Life Sciences, Toyohashi University of Technology, 1-1 Tempaku-cho, Toyohashi, Aichi 441-8580, Japan; iwasa@ens.tut.ac.jp; 6 Ph.D. Program in Chemistry and Ph.D. Program in Biochemistry, The Graduate Center of the City University of New York, New York, NY 10016, USA

**Keywords:** photoluminescent bipyrroles, aldol condensation, organic light emitting diodes, red fluorescence, density functional theory calculation

## Abstract

Photoluminescent divinylbipyrroles were synthesized from 3,3′,4,4′-tetraetyl-2,2′-bipyrrole-5,5′-dicarboxaldehyde and activated methylene compounds via aldol condensation. For mechanistic clarity, molecular structures of Meldrum’s acid- and 1,3-dimethylbarbituric acid-derived divinylbipyrroles were determined by single-crystal X-ray diffraction. Photoluminescent properties of the synthesized divinylbipyrroles in dichloromethane were found to be dependent on the presence of electron withdrawing groups at the vinylic terminal. The divinylbipyrroles derived from malononitrile, Meldrum’s acid, and 1,3-dimethylbarbituric acid showed fluorescent peaks at 553, 576, and 602 nm respectively. Computational studies indicated that the alkyl substituents on the bipyrrole 3 and 3′ positions increased energy level of the highest occupied molecular orbital (HOMO) compared to the unsubstituted derivatives and provided rationale for the bathochromic shift of the ultraviolet-visible (UV-Vis) spectra compared to the previously reported analogs.

## 1. Introduction

**Figure 1:**
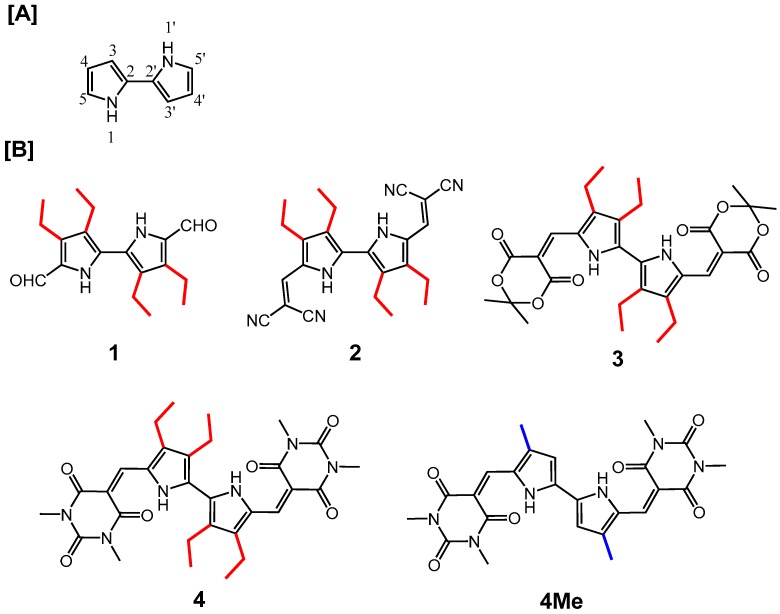
(**A**) Positional numbering system of the bipyrroles; (**B**) Structural formulae of the synthesized divinylbipyrrole compounds **1**–**4** utilized in this study along with the reference compound **4Me**.

Organic photoluminescent materials have attracted much attention in recent years due to their importance in organic light emitting diodes (OLEDs) [1,2,3,4,5] and chemosensors [6,7]. Recently, 2,2′-bipyrrole (Figure 1A), which is also known as a building block of natural red pigments, has been extensively studied because of its luminescent properties and applications in the development of artificial tetrapyrrole and oligopyrrole systems [8,9,10,11,12,13,14,15,16,17,18,19]. Che et al. have demonstrated that a bipyrrole can be a good candidate for the development of luminescent materials of OLEDs [20]. A planar 2,2′-bipyrrole is known to show blue fluorescence upon UV excitation in both solid and solution state. We recently reported the methodology of tuning the fluorescence wavelength of bipyrrole-based fluorophores by utilizing their crystal structure and incorporating suitably placed π-extension [21,22]. In our previous study, a highly efficient photoluminescence was achieved (*λ*_FL_ = 578 nm, *Φ*_FL_ = 88%) by extending π-conjugation, containing a rigid and flat molecular structure, but unfortunately this resulted in poor solubility and crystallinity of the compounds. For practical applications, such as in vivo imaging of a hydrophobic domain of living cells, both longer wavelength fluorescent properties and good solubility are necessary [23,24]. It has been reported that the introduction of vinyl groups into bipyrrole or other chromophores can bathochromically shift the absorption or fluorescence wavelength [25,26,27,28,29,30,31,32]. Furthermore, in synthetic tetrapyrrole chemistry, it has been established that introduction of ethyl or propyl substituents may effectively improve the solubility in organic solvents [12,13].

**Scheme 1:**
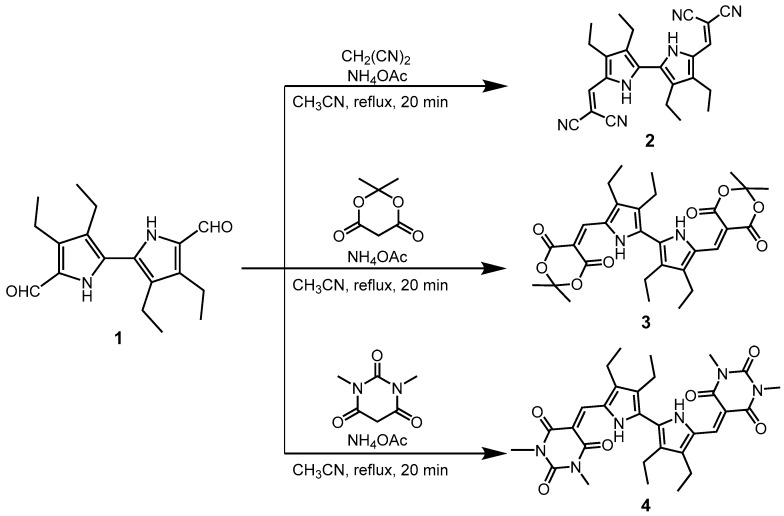
Synthetic scheme of compounds **2**–**4** from **1**.

Herein we report synthesis of novel π-extended bipyrrole derivatives **2**–**4** (Figure 1B) using activated methylene compounds by means of aldol condensation reaction (Scheme 1), study their photophysical properties and utilize time-dependent density functional theory (TD-DFT) calculations to rationalize the desirable bathochromic shifts observed experimentally. It was found that the introduction of four ethyl groups on the bipyrrole 3 and 3′-positions (Figure 1A) resulted in good solubility compared with the previously reported 3,3′-free derivatives [22]. The improvement of the solubility enabled us to obtain single crystals of **3,** and **4,** suitable for X-ray diffraction analysis. Owing to the ethyl substituents at the bipyrrole 3,3′-position, the absorption and fluorescence peaks shifted to the longer wavelength by ca. 20 nm. TD-DFT calculations indicated that the alkyl substituents on the bipyrrole 3,3′-positions destabilize energy level of the highest occupied molecular orbital (HOMO) and resulted in decrease in the HOMO-LUMO gap.

## 2. Results and Discussion

### 2.1. Synthesis

Compound **1** was prepared according to literature method [12]. Novel divinylbipyrroles **2**–**4** bearing four ethyl groups were synthesized from compound **1** and corresponding activated methylene compounds, malononitrile, 2,2-dimethyl-1,3-dioxane-4,6-dione (Meldrum’s acid), and 1,3-dimethylbarbituric acid, by means of an aldol condensation catalyzed by ammonium acetate (Scheme 1) [22]. Crude products were precipitated out from the reaction mixture by the addition of water and collected by filtration. The products were purified by column chromatography using a neutral alumina and eluted with dichloromethane/acetone (95:5, *v*/*v*). Recrystallization of the fraction from dichloromethane and methanol afforded analytically pure crystalline solids of **2**–**4** in 52–80% yield. Each of the synthesized compound exhibited good solubility both in dichloromethane and chloroform.

### 2.2. Nuclear Magnetic Resonance (NMR) Spectroscopy

NMR spectroscopy supported that synthesized divinylbipyrroles derivative (**2**–**4**) have good solubility in chloroform. Although ^13^C NMR spectrum of the previously reported 1,3-dimethylbarbituric acid adduct, **4Me** could not be obtained because of the poor solubility [22], its analog, **4**, afforded clear ^13^C NMR spectrum at similar experimental condition. The ^1^H NMR spectral studies revealed that compounds **3** and **4** have hydrogen bonding between NH and CO. The NH signals of **2**–**4** were observed from 9.5 to 13.7 ppm depending on the functional groups at the vinyl terminal. The large difference in the chemical shift of the NH proton can be explained by strength of the intramolecular hydrogen bonding. Carbonyl groups in compounds **3** and **4** can participate in the intramolecular hydrogen bonding with the NH group and can thus stabilize a seven-membered ring, while cyano group in **2**, which has a linear geometry, is ineffective for such hydrogen bonding interaction.

### 2.3. Crystal Structure

**Figure 2:**
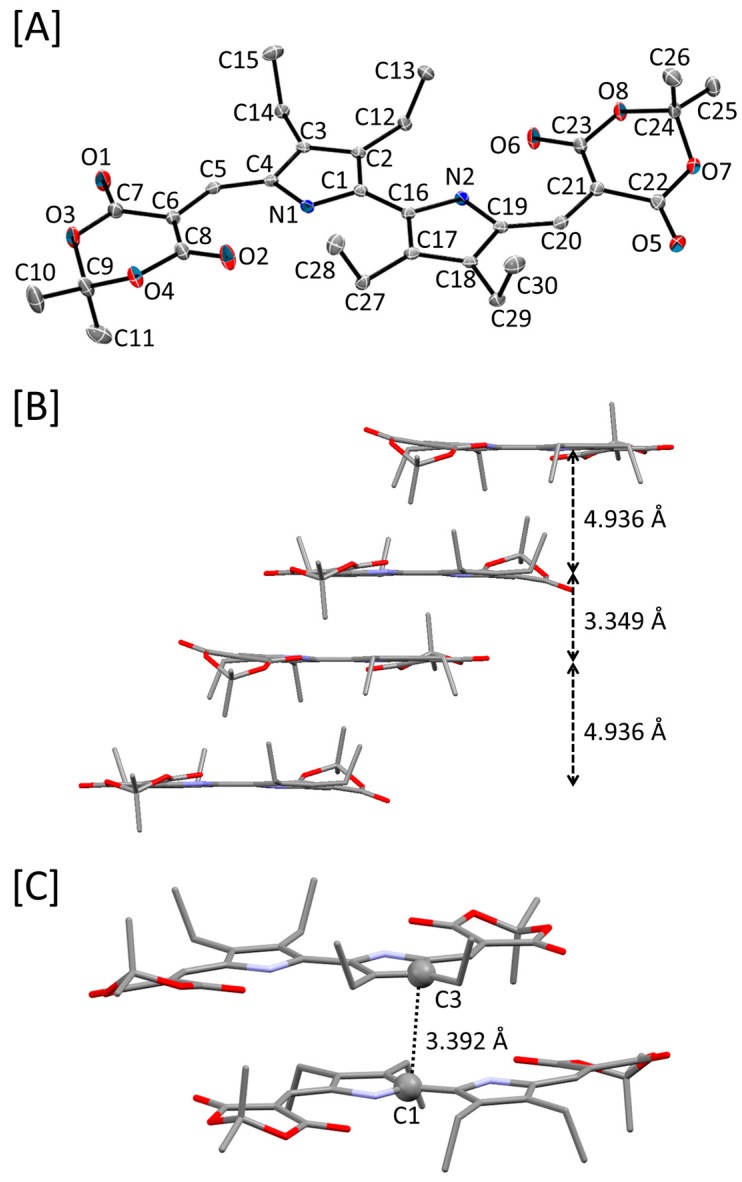
(**A**) The ORTEP diagrams of compound **3** with the atomic labeling; (**B**) Side view with the interlayer distances; (**C**) The closest interatomic distance in a pair of **3**. Hydrogen atoms were omitted for clarity. The thermal ellipsoids were drawn at 50% probability level.

**Figure 3:**
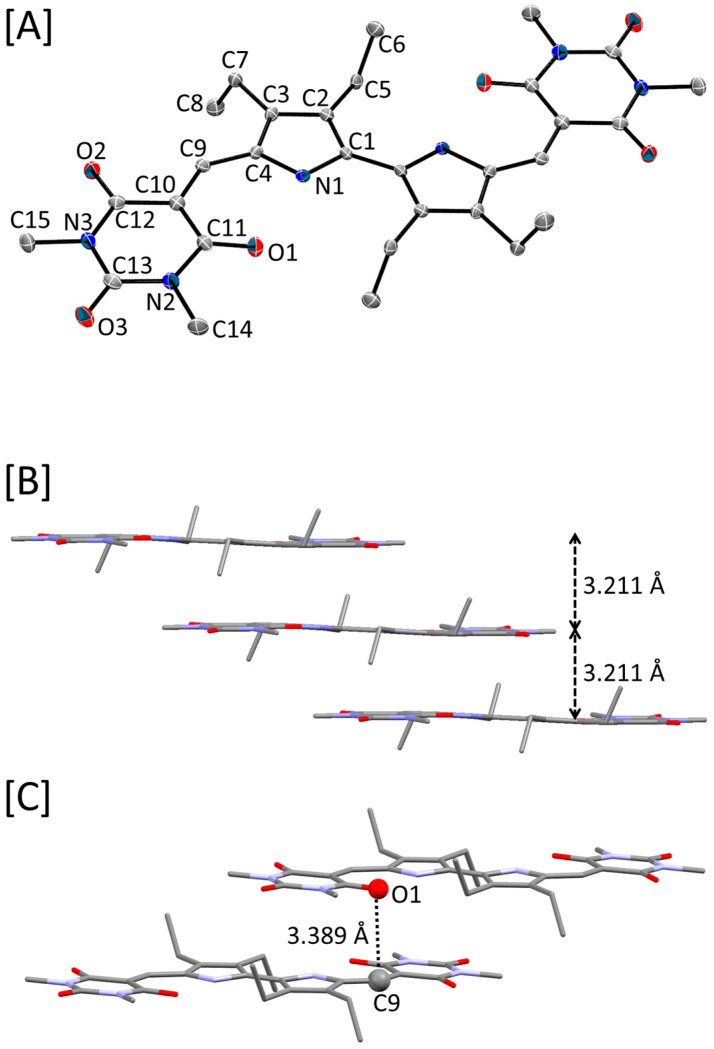
(**A**) The ORTEP diagrams of compound **4** with the atomic labeling; (**B**) side view with the interlayer distances; (**C**) closest interatomic distance in a pair of **4**. Hydrogen atoms were omitted for clarity. The thermal ellipsoids were drawn at 50% probability level.

Compounds **3** and **4** were recrystallized from dichloromethane/methanol and dichloromethane/acetone, respectively. The Oak Ridge Thermal-Ellipsoid Plot Program (ORTEP) diagrams of compounds **3** and **4**, are shown in Figure 2 and Figure 3. Both compounds crystallized in the triclinic space group *P*-1 and showed highly planar structure in the crystals, which is similar to bipyrrole or bithiophene analogs [33,34,35]. Despite this, an extended π-π interaction between bipyrrole rings was interrupted by the relatively hindered ethyl groups. 

Compound **3** has planar structure and all ethyl groups were located at the same side of the bipyrrole plane (Figure 2A). The other side interacted with another molecule to form an antiparallel π-stacked pair. The distance between two mean planes defined by N1, C1, C2, C3, C4, N2, C16, C17, C18, and C19 was 3.349 Å. The second shortest distance between the mean planes were 4.936 Å, which means that the ethyl groups served to block the extended π–π interaction (Figure 2B). The closest interatomic distance between the paired bipyrrole molecules was 3.392 Å (C1 and C3, Figure 2C). Dihedral angle of C23-O6^…^H2-N2 was determined to be 1.84, while C8-O2^…^H1-N1 was 21.44. This suggests that the latter hydrogen bonding is weaker than the former. Geometry around the vinyl group tells us that π-conjugation system effectively spreads from one vinyl group to the other. The carbon–carbon distances, C5–C6 and C20–C21 were found to be 1.389 and 1.382 Å, respectively. A. Mendoza and F.-F. Jian independently reported crystal structures of related Meldrum’s acid adducts [36,37,38]. In the literature, when a vinyl and a phenyl group twist each other, the carbon–carbon double bond at the vinyl group becomes shorter (1.33–1.34 Å). In our case however, π-conjugation system extension was successfully achieved by introducing the vinyl group due to the intramolecular hydrogen bonding interaction.

In compound **4**, the four ethyl groups were located on both sides of the bipyrrole plane (Figure 3A). The interlayer distance between the mean planes defined by N1, C1, C2, C3, and C4 was determined to be 3.211 Å, but direct interaction between the pyrrole rings was not observed (Figure 3B). Instead, there is another intermolecular interaction between a carbonyl group of barbituric unit (O1) and an electron deficient vinyl group (C9) of the neighboring molecule (Figure 3C) [21,39,40,41]. Compound **4** also showed strong intramolecular hydrogen bonding between the carbonyl oxygen atom and the NH groups. In addition, the dihedral angle of C11-O1^…^H1-N1 were 2.36, which means that **4** was more planar than **3** in the solid state. This is probably because of the hybridization of the terminal six membered ring. The vinyl terminal in **3** includes a sp^3^ carbon while **4** has all sp^2^ hybridization. Thus, the hydrogen bonding geometry in **4** becomes nearly flat.

### 2.4. UV-Vis Absorption and Photoluminescence

**Figure 4:**
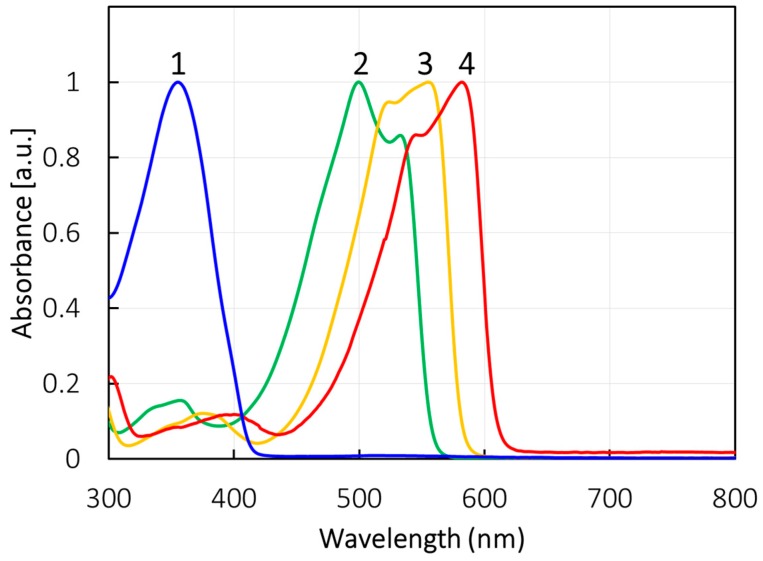
Absorption spectra of **1**–**4** in dichloromethane. The absorption spectra were normalized at the maxima.

**Figure 5:**
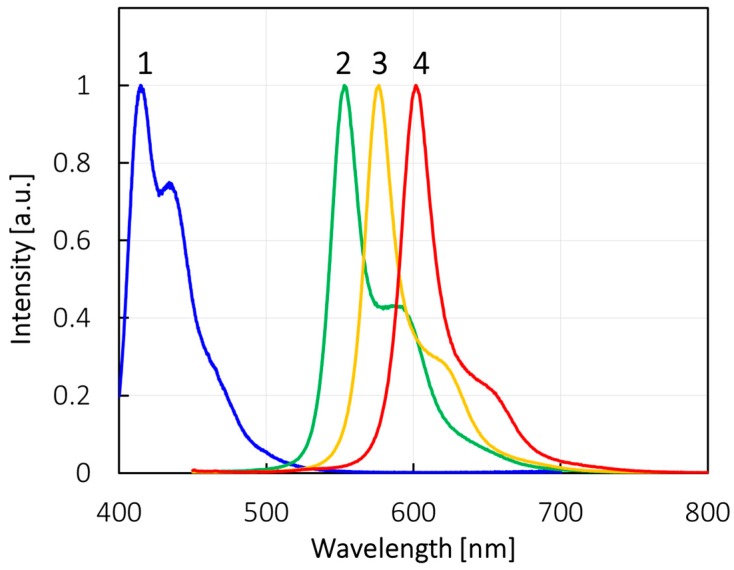
Fluorescence spectra of **1**–**4** in dichloromethane. The excitation wavelength was fixed at 365 nm in all scans.

Bipyrrole **2**–**4** showed large bathochromic shift of absorption and fluorescence compared to the starting compound **1** and depended on the structure of the terminal substituents. In particular, compound **4** having the barbituric unit displayed the fluorescence maxima at 602 nm. Absorption and fluorescence spectra and photophysical parameters are shown in Figure 4 and Figure 5, and Table 1, respectively. These results suggest that expansion of the π-conjugation system by introduction of the vinyl groups effectively influenced the absorption and luminescent properties. Interestingly, absorption and fluorescence maxima of **4** shifted to the longer wavelength compared to the previously reported analog, **4Me**, by ca. 20 nm.

Absolute quantum yield measurements revealed that the cyclic and rigid terminal structure led to highly efficient photoluminescence (Table 1). Compound **2** showed the lowest quantum yield (5%) probably because of molecular flexibility. Even though the present compounds have four alkyl groups on the bipyrrole periphery, they did not influence on the quantum yields negatively. The quantum yields of compounds **3** and **4** were determined to be 81 and 93%, respectively. These results support the notion that both a rigid cyclic structure and intramolecular hydrogen bonding play an important role in achieving the high quantum yield for bipyrrole systems. Although alkyl groups are known to promote the internal conversion, our present tetraethylbipyrrole system have comparable quantum yield with the previously reported dialkyl analog such as **4Me**.

### 2.5. TD-DFT Calculations

**Figure 6:**
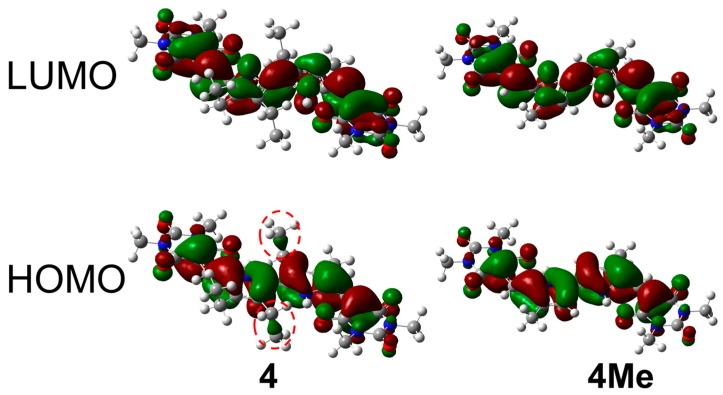
LUMO (**top**) and HOMO (**bottom**) orbitals (isovalue of 0.02) of compounds **4** and **4Me**. The red and green surfaces show positive and negative wave functions, respectively. The broken red circles show ethyl groups at the 3 and 3′ positions.

To rationalize the differences observed in the absorption wavelength, TD-DFT calculations were performed using the Gaussian 09 suite of programs [42]. The initial atomic coordinates for the compounds are shown in the Supplementary Materials. The structures were optimized and evaluated using the CAM-B3LYP/6-31G(d) [43] level. The solvent effect was considered in dichloromethane by the polarizable continuum model using the integral equation formalism variant (IEFPCM) [44]. The calculated absorption maxima are summarized in Table 2. The TD-DFT calculation supported the absorption spectral difference between **4** and **4Me**. On the other hand, calculated optical properties of **2** did not agree the experimental results. Relatively flexible compound **2** can take many conformation in the solution thus the experimental absorption spectra likely reflects the average sum of the various possible conformations. The calculation also provided the insight into the differences between the “tetra-alkylated” and “di-alkylated” bipyrroles. Figure 6 shows the HOMO and the LUMO orbitals of **4** and **4Me**. It is clear from this that an ethyl group on the pyrrole 3,3′-positions contributes to the HOMO orbital. The HOMO level of **4** was 0.12 eV higher than that of **4Me** due to the electron donation from the methyl group. On the other hand, there was no obvious difference between the LUMO orbitals in **4** and **4Me**. This result explains the bathochromic shift of absorption and fluorescence wavelength in **4** compared to **4Me**.

The vinyl groups and attached electron withdrawing groups significantly contributed to HOMO and LUMO. Thus, the TD-DFT calculations revealed that the alkyl groups on the bipyrrole 3,3′-positions greatly influenced photophysical properties.

## 3. Experimental

### 3.1. Materials and Instruments

All chemical reagents and solvents used in this study were obtained from commercial sources and used as received unless otherwise stated. 3,3′,4,4′-tetraethyl-5,5′-diformyl-2,2′-bipyrrole (**1**) has been prepared according to the literature [12]. Compounds **2**–**4** were synthesized using the similar reaction condition reported in the previous paper [22].

UV-Vis absorption spectra were recorded on a JASCO V-670 spectrophotometer (JASCO Corporation, Tokyo, Japan). Emission spectra were measured by a Hitachi F7000 spectrophotometer (Hitachi High-Technology, Tokyo, Japan). ^1^H and ^13^C NMR spectra were obtained at 25 °C on a JEOL ECS-400 FT-NMR spectrometer (JEOL, Tokyo, Japan) with tetramethylsilane as an internal standard of the chemical shift. Infrared (IR) spectra were recorded on a JASCO FT/IR-410 spectrometer (JASCO Corporation, Tokyo, Japan). Electrospray ionization time of flight mass (ESI-TOF-MS) spectra were measured with JEOL JMS-T100CS spectrometer (JEOL, Tokyo, Japan) using methanol/dichloromethane as a solvent system. The drying cavity was heated to 250 °C. The needle voltage, orifice 1, and orifice 2 voltages were within the range of 2500–4000 V, 50–80 V, and 3–15 V, respectively. Absolute photoluminescent quantum yields of **2**–**4** were determined by using a Hamamatsu Absolute PL Quantum Yield Spectrometer C11347 Quantaurus-QY (400–1100 nm), which equipped with an integrating sphere.

### 3.2. X-ray Crystallography

X-ray crystallography was performed using a Bruker SMART APEX CCD diffractometer equipped with graphite-monochromated Mo Kα radiation (λ = 0.71073 Å) from a fine-focus sealed tube operated at 50 kV and 30 mA. Single crystals of **3** and **4** were mounted on a glass fiber and the data frames were integrated using SAINT [45]. The integrated data were merged to give a unique data set for the structure determination. Absorption corrections by SADABS were carried out [46]. The structure was solved by a direct method and refined by the full-matrix least-squares method on all F^2^ data using the SHELXL-2014/7 suite of programs [47]. All nonhydrogen atoms were anisotropically refined. Hydrogen atoms were placed at geometrically idealized positions and constrained to ride on their parent atoms with U_iso_(H) = 1.2U_eq_(NH, CH and CH_2_) and U_iso_(H) = 1.5U_eq_(CH_3_). Crystallographic data (excluding structure factors) have been deposited with the Cambridge Crystallographic Data Centre as supplementary publication no. CCDC-1535686 and 1535687 for **3** and **4**, respectively. Copies of the data can be obtained free of charge on application to CCDC, 12 Union Road, Cambridge CB21EZ, UK (fax: (+44) 1223-336-033; email: deposit@ccdc.cam.ac.uk).

### 3.3. Synthesis

*(a) Compound* **2**. In a round bottomed flask, 32 mg (0.11 mmol) of **1** and 311 mg (4.7 mmol) of malononitrile were dissolved in 10 mL of acetonitrile. The solution was heated to refluxing temperature and then 359 mg (4.7 mmol) of ammonium acetate was added to the solution. The reaction mixture turned dark red and was further heated for 20 min. The reaction mixture was cooled to room temperature and 20 mL of water was added to the mixture. Resulting red precipitates were collected by filtration and washed with water. The product was purified by a neutral alumina. Yield: 29 mg (69%). Elemental Analysis Found: C 72.44, H 6.08, N 20.96, Calcd. (C_24_H_24_N_6_) C 72.70, H 6.10, N 21.20; ^1^H NMR (400 MHz, 298 K, CDCl_3_, ppm): δ = 9.57 (s, 2H, NH), 7.47 (s, 2H, vinyl-CH), 2.72, 2.66 (q, *J* = 7.5 Hz, 8H, -CH_2_CH_3_), 1.21, 1.16 (t, *J* = 7.5 Hz, 12H, -CH_2_CH_3_); ^13^C NMR (100 MHz, 298 K, CDCl_3_, ppm) δ = 142.2, 140.3, 130.1, 128.6, 125.6, 116.3, 115.0, 68.4, 17.9, 17.5, 17.0, 15.5; IR (KBr) wavenumber cm^−1^: 2217 (ν_CN_); ESI-TOF-MS (CH_3_OH/CH_2_Cl_2_): *m*/*z* = 397 ([M + H]^+^).

*(b) Compound* **3**. Yield: 80%. Elemental Analysis Found: C 64.43, H 6.43, N 4.96, Calcd. (C_30_H_36_N_2_O_8_) C 65.20, H 6.57, N 5.07; ^1^H NMR (400 MHz, 298 K, CDCl_3_, ppm): δ = 12.96 (s, 2H, NH), 8.28 (s, 2H, vinyl-CH), 2.85, 2.82 (q, *J* = 7.5 Hz, 8H, -CH_2_CH_3_), 1.79 (s, 12H, C(CH_3_)_2_), 1.26, 1.20 (t, *J* = 7.5 Hz, 12H, -CH_2_CH_3_); ^13^C NMR (100 MHz, 298 K, CDCl_3_, ppm) δ = 165.0, 164.5, 145.4, 137.7, 132.4, 130.6, 127.9, 104.3, 99.1, 77.2, 27.2, 18.1, 17.8, 17.3, 15.6; IR (KBr) wavenumber cm^−1^: 1731, 1681 (ν_CO_); ESI-TOF-MS (CH_3_OH/CH_2_Cl_2_): *m*/*z* = 552 ([M]^+^); X-ray crystallography: C_30_H_36_N_2_O_8_, triclinic, P−1, a = 10.006(14) Å, b = 11.205(16) Å, c = 13.232(19) Å, α = 82.71(3)°, β = 80.50(5)°, γ = 73.03(3)°, V = 1395(3) Å^3^, Z = 2, T = 100 K, λ(Mo Kα) = 0.096 mm^−1^, R1 = 0.0428 (I > 2σ(I)), wR2 = 0.1091, GOF = 1.363.

*(c) Compound* **4**. Yield: 52%. Elemental Analysis Found: C 62.19, H 6.18, N 14.37, Calcd. (C_30_H_36_N_6_O_6_) C 62.49, H 6.29, N 14.57; ^1^H NMR (400 MHz, 298 K, CDCl_3_, ppm): δ = 13.65 (s, 2H, NH), 8.38 (s, 2H, vinyl-CH), 3.43 (s, 12H, -NCH_3_), 2.91, 2.86 (q, *J* = 7.5 Hz, 8H, -CH_2_CH_3_), 1.28, 1.22 (t, *J* = 7.5 Hz, 12H, -CH_2_CH_3_); ^13^C NMR (100 MHz, 298 K, CDCl_3_, ppm) δ = 164.0, 163.6, 151.6, 145.0, 137.2, 132.1, 130.7, 128.7, 104.7, 28.8, 28.4, 18.2, 17.9, 17.5, 15.7; IR (KBr) wavenumber cm^−1^: 1720, 1660, 1638 (ν_CO_); ESI-TOF-MS (CH_3_OH/CH_2_Cl_2_): *m*/*z* = 577 ([M + H]^+^); X-ray crystallography: C_30_H_36_N_6_O_6_, triclinic, P−1, a = 8.363(9) Å, b = 8.754(11) Å, c = 10.446(12) Å, α = 102.81(2)°, β = 108.034(14)°, γ = 100.24(4)°, V = 683.6(14) Å^3^, Z = 1, T = 100 K, λ(Mo Kα) = 0.099 mm^−1^, R1 = 0.0572 (I > 2σ(I)), wR2 = 0.1468, GOF = 1.027.

### 3.4. Computational Methods

The computational studies were carried out by using the Gaussian 09 suite of program [42]. The initial coordinates of the compounds were optimized by the CAMB3LYP/6-31G(d) basis set [43]. The calculations were performed in dichloromethane. The solvent environment was treated using the IEFPCM model [44]. The transition energies were also calculated by using the CAMB3LYP/6-31G(d) level of theory.

## 4. Conclusions

In conclusion, we synthesized novel luminescent bipyrroles with four ethyl groups and vinylic extensions. The ethyl groups were found to be effective for the solubility improvement. The X-ray structure analysis revealed that compound **3** and **4** have a planar structure and possess intramolecular hydrogen bonding in the solid state. The strong hydrogen bonding resulted in a rigid molecular structure, which led to enhanced quantum yield. The ethyl substituents served to not only provide solubility improvement in organic solvents but also in tuning of the HOMO–LUMO gap. Compound **4**, for example, showed 13 nm longer absorption wavelength and exhibited enhanced photoluminescence compared to the previously reported dimethyl analog **4Me**. TD-DFT calculation further shed light that the alkyl substitutions at the bipyrrole 3 and 3′-positions have an effect on the HOMO energy level, thereby resulting in the bathochromic shift of the absorption and fluorescence spectra. It is anticipated that the knowledge gained from these studies will contribute towards the development of enhanced organic photoluminescent materials utilizing π-extended bipyrrole derivatives. 

## Figures and Tables

**Table 1:** Photophysical parameters for **1**–**4** and **4Me**.

Compounds	*λ*_Abs_ (nm)	*λ*_FL_ (nm)	*Φ*_FL_ (−)
**1**	355	414	NA ^a^
**2**	499, 531	553	0.05
**3**	555	576	0.81
**4**	582	602	0.93
**4Me** ^[22]^	569	578	0.88

^a^ Could not be obtained due to the wavelength limit.

**Table 2:** UV-Vis spectroscopic data and calculated lowest excited states of **1**–**4** and **4Me** compounds.

Compounds	*λ*_Abs_ (Obs.) (nm)	*λ*_Abs_ (Calcd.) (nm)	Transitions	Oscillator Strength
**1**	355	403.31	81(HOMO)‒82(LUMO)	1.0825
**2**	499, 531	556.11	104(HOMO−1)‒107(LUMO+1) 105(HOMO)‒106(LUMO)	1.5891
**3**	555	553.40	146(HOMO−1)‒149(LUMO+1) 147(HOMO)‒148(LUMO)	1.7047
**4**	582	574.31	152(HOMO−1)‒155 (LUMO+1) 153(HOMO)‒154(LUMO)	1.8560
**4Me**	569	560.00	128(HOMO−1)‒131(LUMO+1) 129(HOMO)‒130(LUMO)	1.9396

## Supplementary Materials

Supplementary materials are available online. Crystallographic data for **3** and **4**, ^1^H NMR. ^13^C NMR, IR and MS spectra, elemental analysis of **2**–**4**, absolute quantum yield measurements of **2**–**4**, HOMO and LUMO orbitals of **1**–**4** and **4Me**, and initial coordinates of **1**–**4** and **4Me** for TD-DFT calculations.
