# Supplementary material for: Synthesis, Crystal Structure, and Photoluminescent Properties of 3,3′,4,4′-Tetraethyl-5,5′-divinyl-2,2′-bipyrrole Derivatives
Source: Molecules. 2017 Oct 26;22(11):1816. doi: 10.3390/molecules22111816 (PMC6150395; doi:10.3390/molecules22111816)
Supplement: Supplementary file 1 [file molecules-22-01816-s001.pdf]

Supplementary Materials

Synthesis, Crystal Structure, and Photoluminescent Properties of 3,3′,4,4′-Tetraethyl-5,5′-divinyl-2,2′-bipyrrole Derivatives

Toru Okawara [1,*], Reo Kawano [2], Hiroya Morita [2], Alan Finkelstein [3], Renjiro Toyofuku [4], Kanako Matsumoto [4], Kenji Takehara [1], Toshihiko Nagamura [1], Seiji Iwasa [5], and Sanjai Kumar [3]

[1] Department of Creative Engineering, National Institute of Technology, Kitakyushu College, 5-20-1 Shi-i, Kokuraminami-ku, Kitakyushu 802-0985, Japan
[2] Advanced School of Creative Engineering, National Institute of Technology, Kitakyushu College, 5-20-1 Shi-i, Kokuraminami-ku, Kitakyushu 802-0985, Japan
[3] Department of Chemistry and Biochemistry, Queens College, Queens New York 11367, USA; Ph.D. Program in Chemistry and Ph.D. Program in Biochemistry, The Graduate Center of the City University of New York, New York, New York 10016, USA
[4] Department of Materials Science and Chemical Engineering, National Institute of Technology, Kitakyushu College, 5-20-1 Shi-i, Kokuraminami-ku, Kitakyushu 802-0985, Japan
[5] Department of Environmental and Life Sciences, Toyohashi University of Technology, 1-1 Tempaku-cho, Toyohashi, Aichi 441-8580, Japan

* Correspondence: okawara@kct.ac.jp; Tel.: +81-93-964-7300

Table of contents

Page. 2−4	Crystallographic data and geometrical parameters for **3** and **4**.
Page. 5−8	Analysis of **2**.
Page. 9−12	Analysis of **3**.
Page. 13−16	Analysis of **4**.
Page. 17	Elemental analysis of **2-4**.
Page. 18-20	Absolute quantum yield measurements of **2-4**.
Page. 21	HOMO and LUMO orbital diagrams of the compounds.
Page. 22−33	The initial coordinates for the TD-DFT calculations.

Table S1. Crystallographic data for **3** and **4**.

	**3**	**4**
Chemical formula	$C_{30}H_{36}N_2O_8$	$C_{30}H_{36}N_6O_6$
Formula weight	552.61	576.65
Temperature [K]	100	100
Wavelength [Å]	0.71073	0.71073
Crystal system	Triclinic	Triclinic
Space group	*P*−1	*P*−1
a [Å]	10.006(14)	8.363(9)
b [Å]	11.205(16)	8.754(11)
c [Å]	13.232(19)	10.446(12)
α [°]	82.71(3)	102.81(2)
β [°]	80.50(5)	108.034(14)
γ [°]	73.03(3)	100.24(4)
Volume [$Å^3$]	1395(3)	683.6(14)
Z	2	1
Density (calculated) [g/cm^3]	1.316	1.401
Absorption coefficient [mm^{-1}]	0.096	0.099
F(000)	588	306
θ [°]	1.57 to 26.50	2.15 to 26.50
Reflections collected	8408	3951
Independent reflections	5733 [$R_{(int)}$ = 0.0222]	2660 [$R_{(int)}$ = 0.0320]
Data / restraints / parameters	5733 / 0 / 369	2660 / 0 / 194
Completeness	99.2%	98.8%
Goodness-of-fit on F^2	1.363	1.027
$R1^a$ [$I>2\sigma(I)$]	0.0428	0.0572
$wR2^b$ (all data)	0.1091	0.1468
Largest diff. peak and hole [e.$Å^{-3}$]	0.331 and −0.253	0.331 and −0.282

[a] $R1 = (\sum||F_o|-|F_c||)/(\sum|F_o|)$. [b] $wR2 = [\sum w(F_o^2 - F_c^2)^2/\sum wF_o^4]^{1/2}$, where $w = 1/[\sigma^2(F_o^2)+(0.0442(F_o^2+2F_c^2)/3)^2]$ for **3** and $1/[\sigma^2(F_o^2)+ (0.0551(F_o^2+2F_c^2)/3)^2+0.4325(F_o^2+2F_c^2)/3]$ for **4**.

Table S2. Bond lengths for **3**.

Lengths [Å]			
N1-C1	1.366(2)	C18-C19	1.418(3)
N1-C4	1.385(2)	C18-C29	1.511(2)
N2-C16	1.370(2)	C19-C20	1.420(2)
N2-C19	1.384(2)	C20-C21	1.382(3)
C1-C2	1.430(2)	C21-C22	1.483(2)
C1-C16	1.459(2)	C21-C23	1.459(2)
C2-C3	1.405(2)	C24-C25	1.514(3)
C2-C12	1.512(3)	C24-C26	1.522(3)
C3-C4	1.426(3)	C27-C28	1.541(2)
C3-C14	1.511(2)	C29-C30	1.538(3)
C4-C5	1.414(2)	O1-C7	1.214(2)
C5-C6	1.389(3)	O2-C8	1.224(2)
C6-C7	1.483(2)	O3-C7	1.372(3)
C6-C8	1.460(2)	O3-C9	1.437(2)
C9-C10	1.519(3)	O4-C8	1.366(2)
C9-C11	1.515(3)	O5-C22	1.214(2)
C12-C13	1.542(3)	O6-C23	1.225(2)
C14-C15	1.536(3)	O7-C22	1.371(3)
C16-C17	1.430(2)	O7-C24	1.439(2)
C17-C18	1.406(2)	O8-C23	1.360(2)
C17-C27	1.511(3)	O8-C24	1.456(2)

Table S3. Bond lengths for **4**.

Lengths [Å]			
N1-C1	1.368(4)	C3-C4	1.420(4)
N1-C4	1.388(3)	C3-C7	1.513(4)
N2-C11	1.402(4)	C4-C9	1.411(4)
N2-C13	1.404(4)	C5-C6	1.537(4)
N2-C14	1.481(4)	C7-C8	1.538(4)
N3-C12	1.393(4)	C9-C10	1.389(4)
N3-C13	1.387(4)	C10-C11	1.451(4)
N3-C15	1.481(4)	C10-C12	1.485(4)
C1-C1[a]	1.460(5)	O1-C11	1.241(3)
C1-C2	1.426(4)	O2-C12	1.230(4)
C2-C3	1.403(4)	O3-C13	1.218(3)
C2-C5	1.510(4)		

[a] Symmetry operation (1−*x*, 2−*y*, 1−*z*)

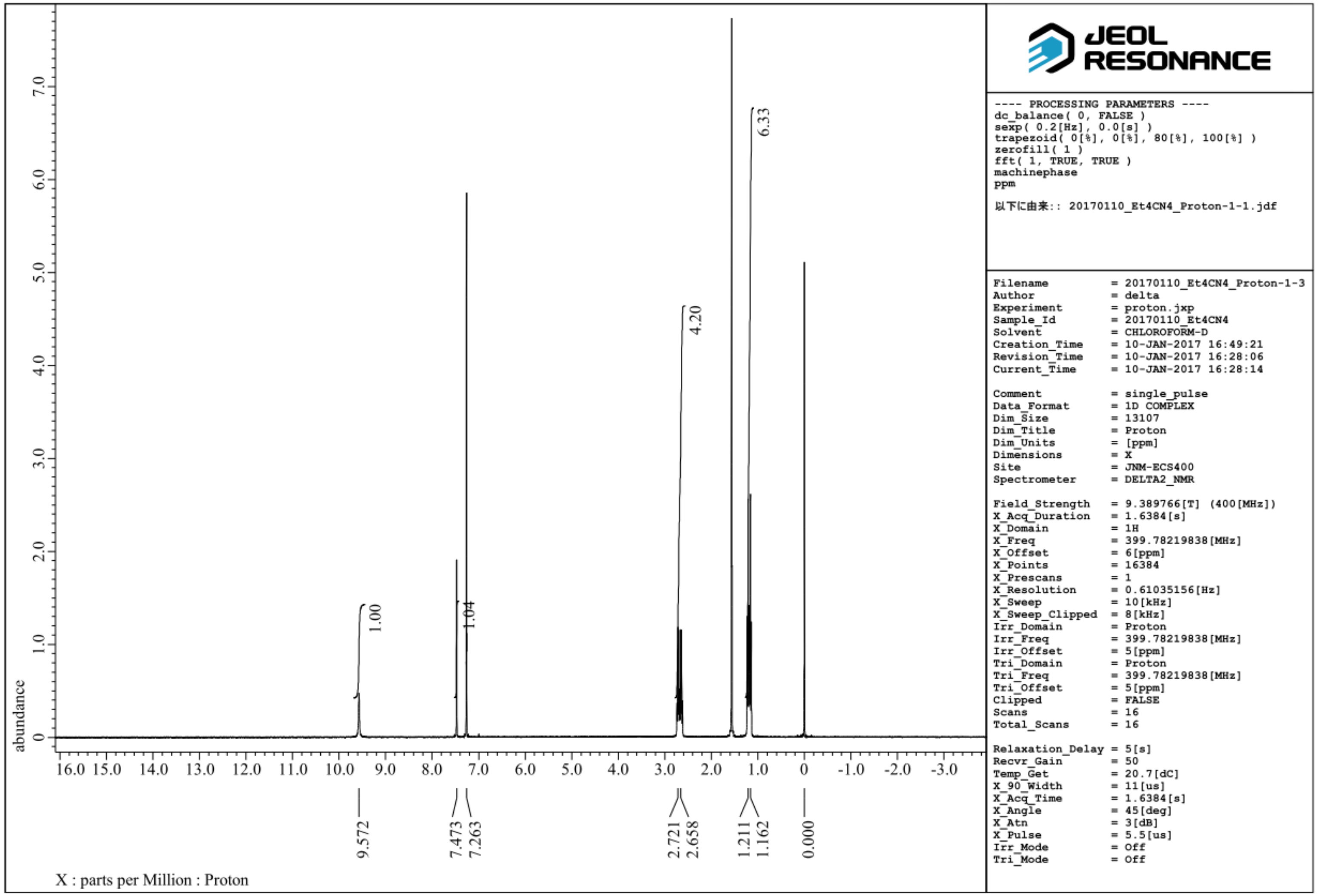

Figure S1. ^{1}H NMR spectrum of **2** in $CDCl_3$ at 25°C.

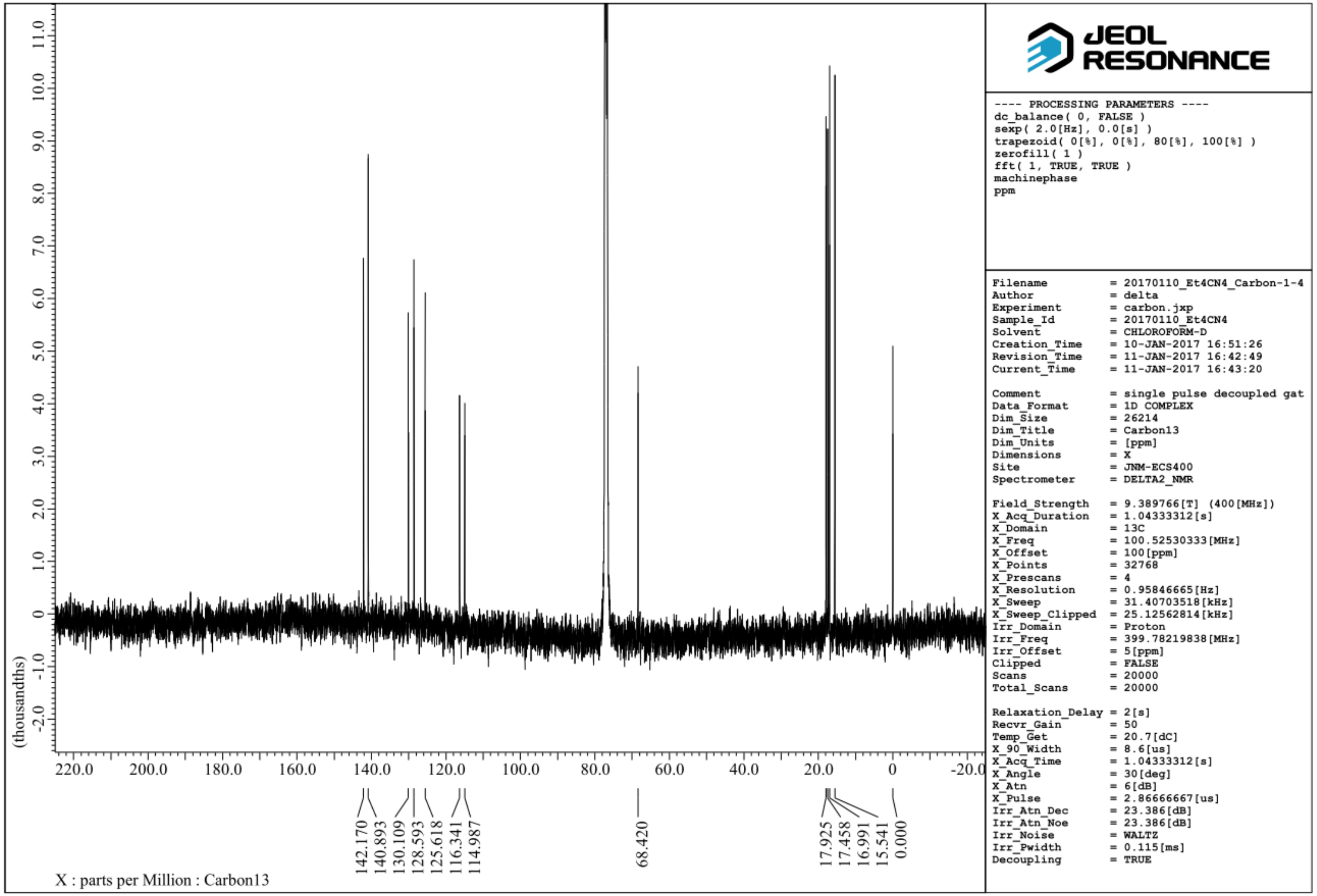

Figure S2. ^{13}C NMR spectrum of **2** in $CDCl_3$ at 25°C.

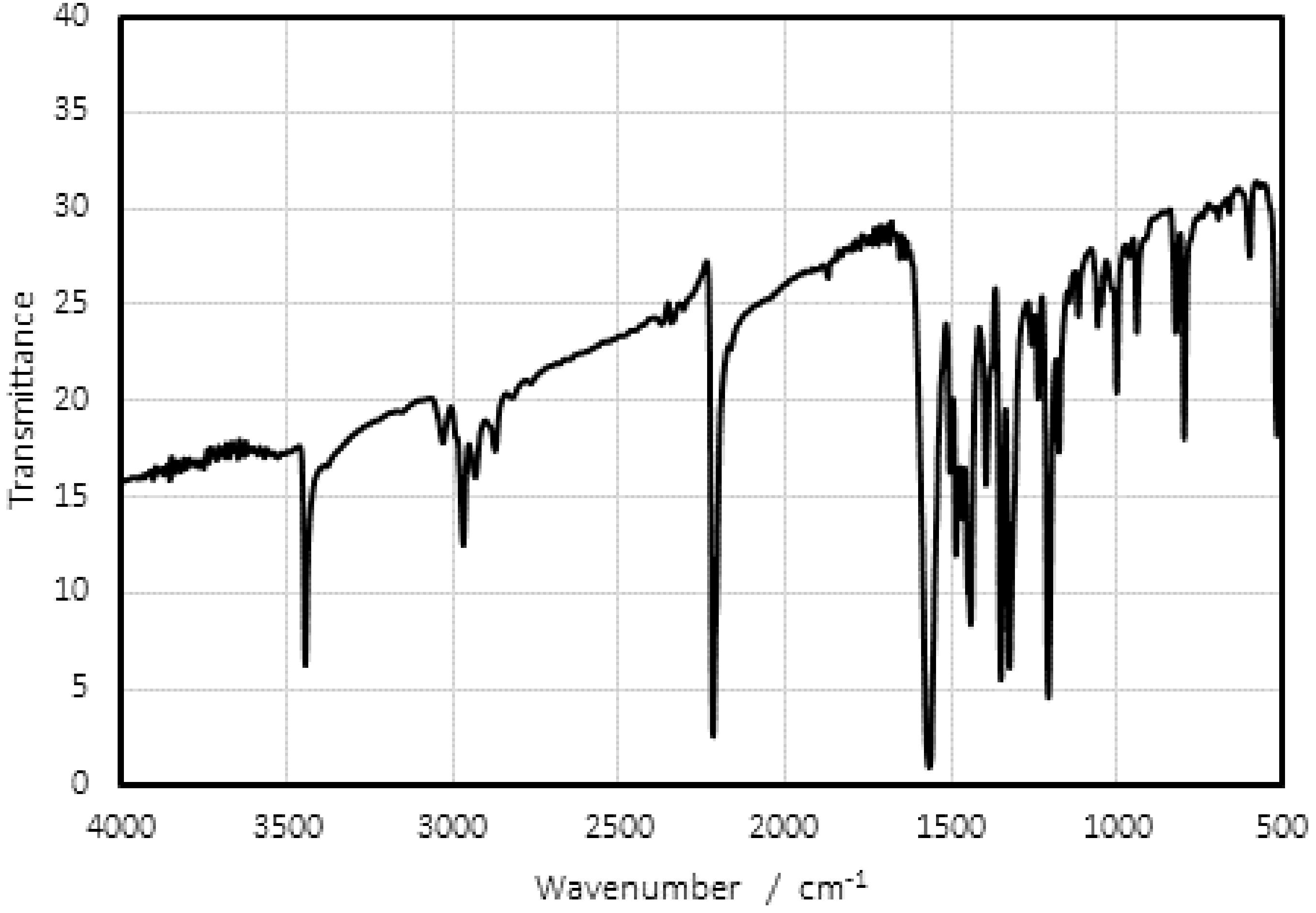

Figure S3. IR spectrum of **2**.

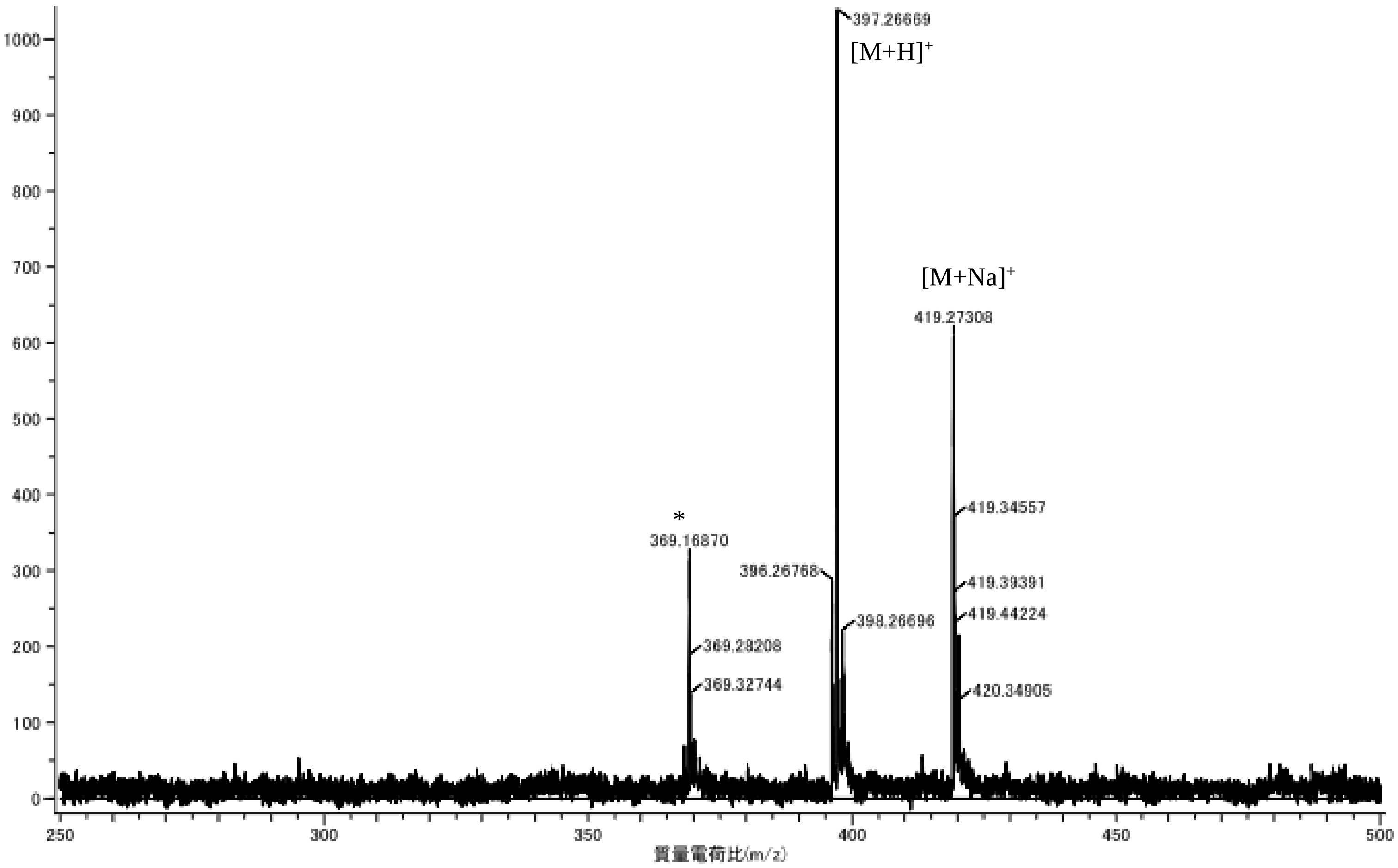

Figure S4. ESI-TOF-MS spectrum of **2** in CH_2Cl_2/CH_3OH. *Impurity in the solvents.

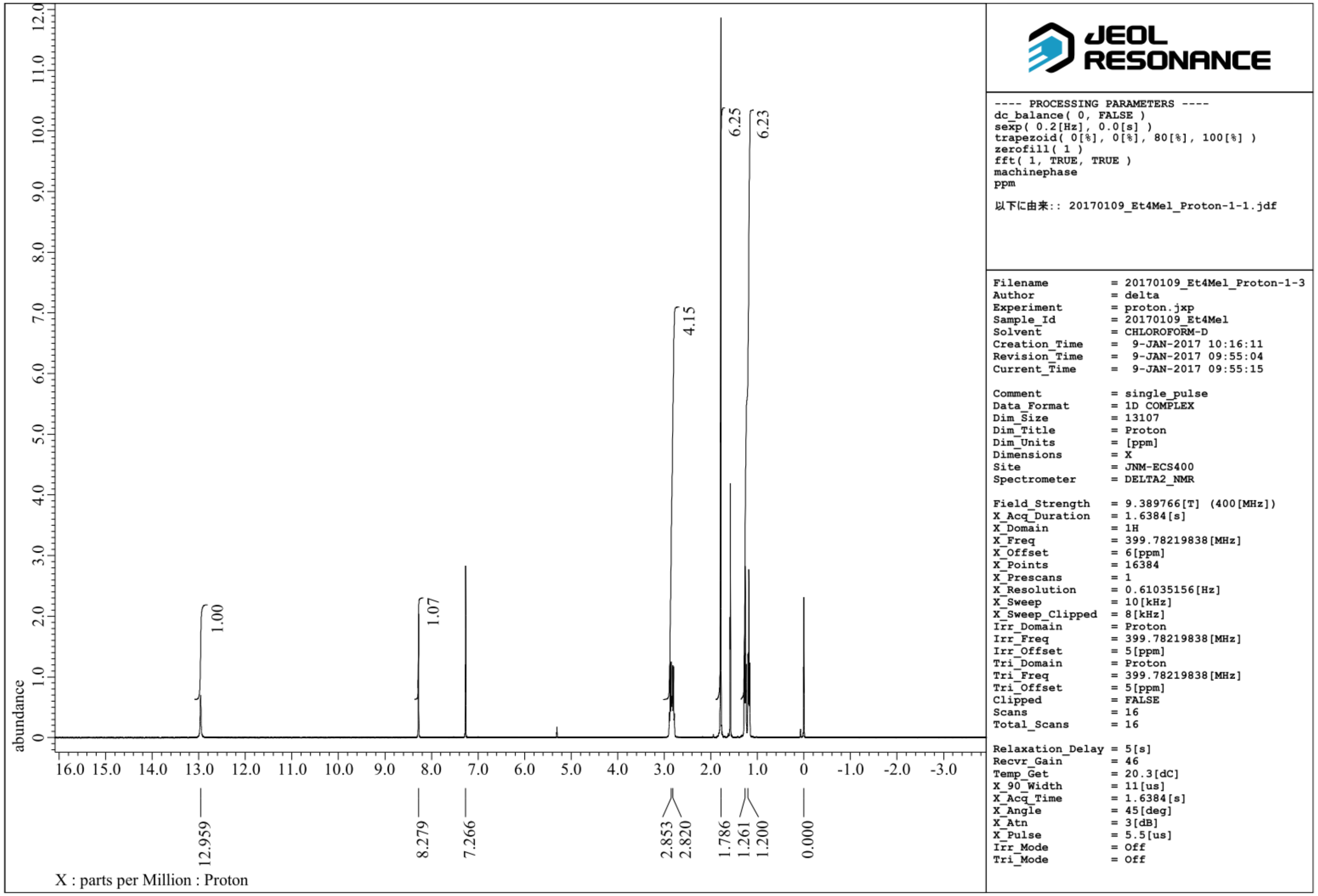

Figure S5. ^{1}H NMR spectrum of **3** in $CDCl_3$ at 25°C.

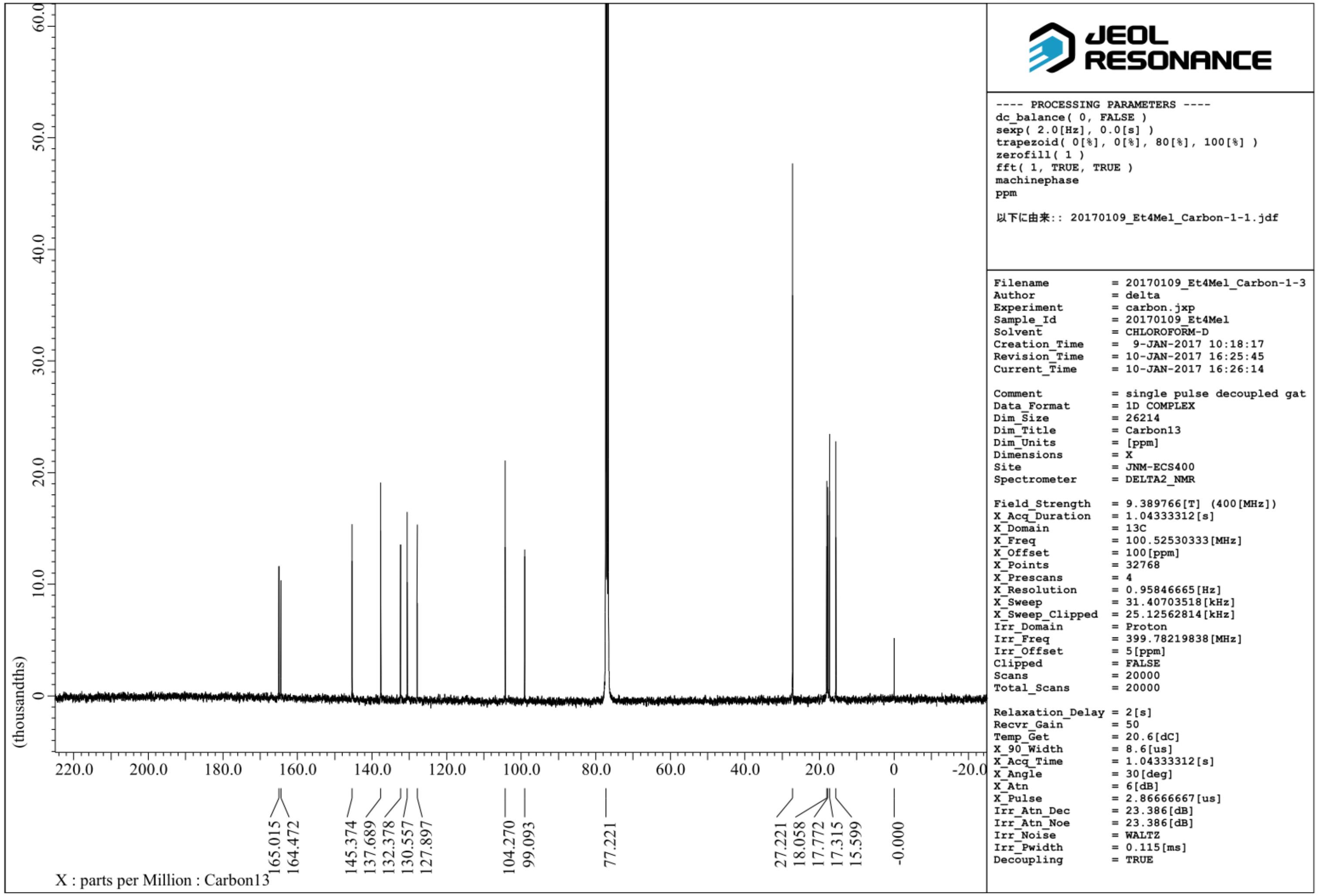

Figure S6. ^{13}C NMR spectrum of **3** in $CDCl_3$ at 25°C.

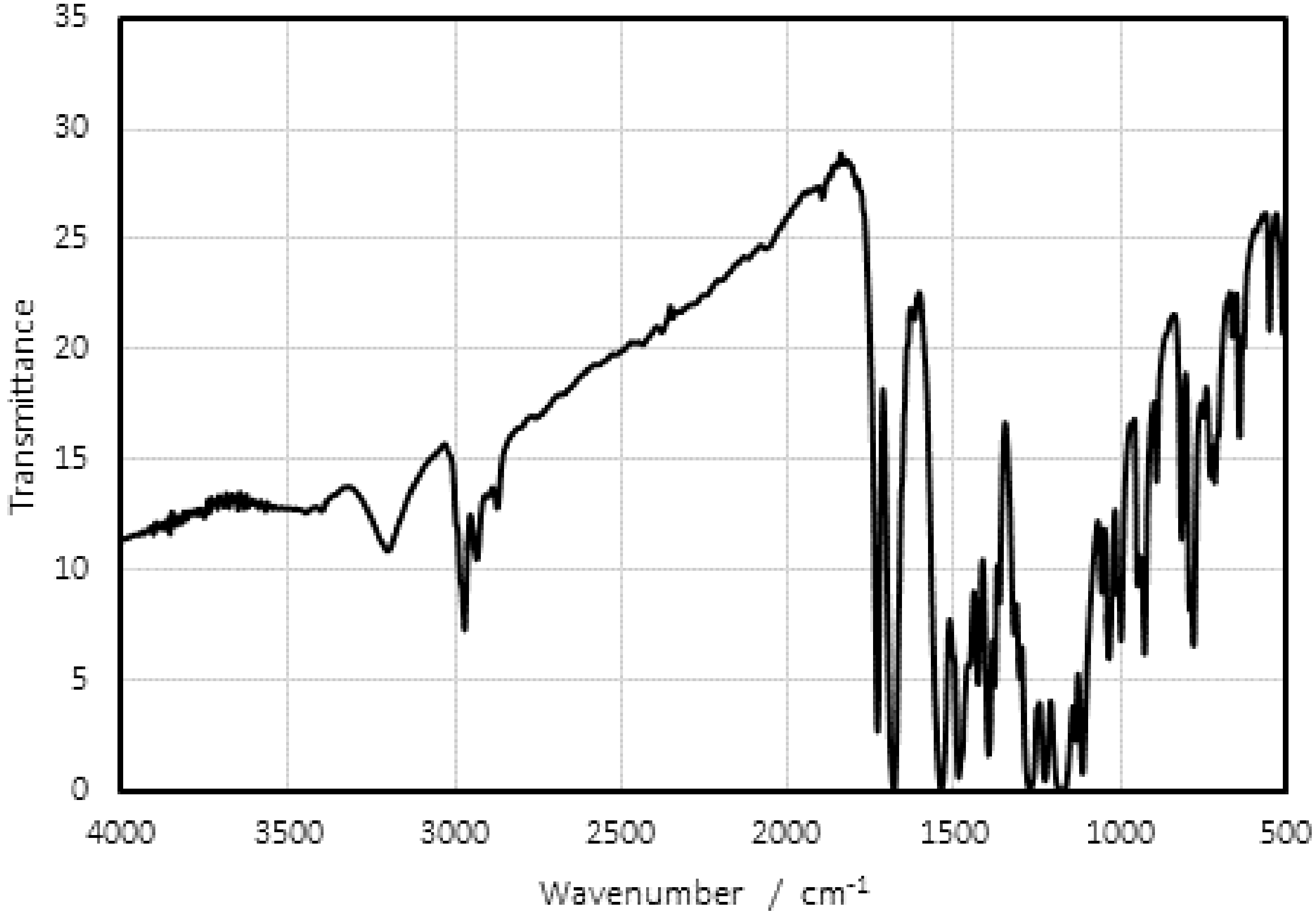

Figure S7. IR spectrum of **3**.

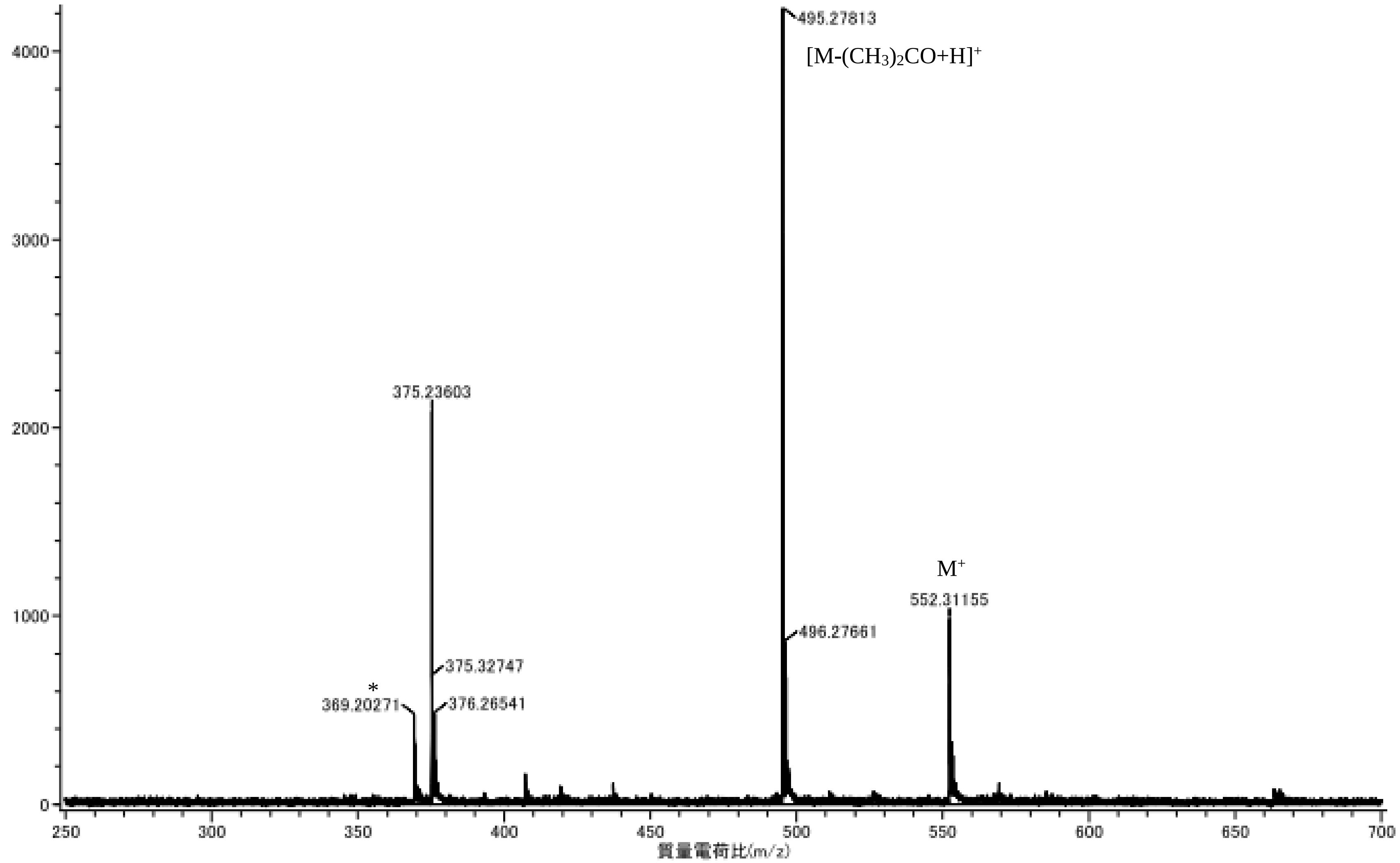

Figure S8. ESI-TOF-MS spectrum of **3** in CH_2Cl_2/CH_3OH. *Impurity in the solvents.

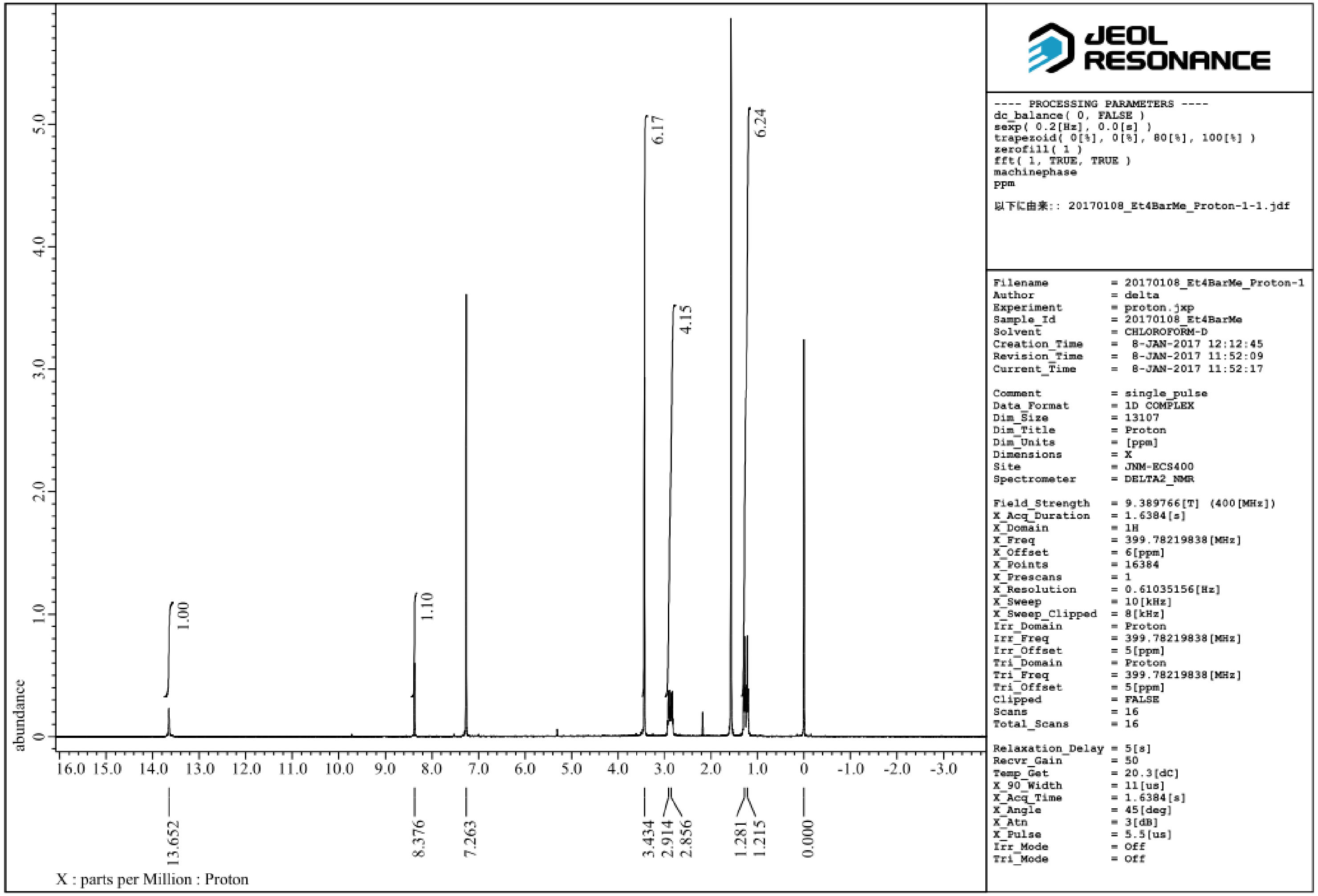

Figure S9. ^{1}H NMR spectrum of **4** in $CDCl_3$ at 25°C.

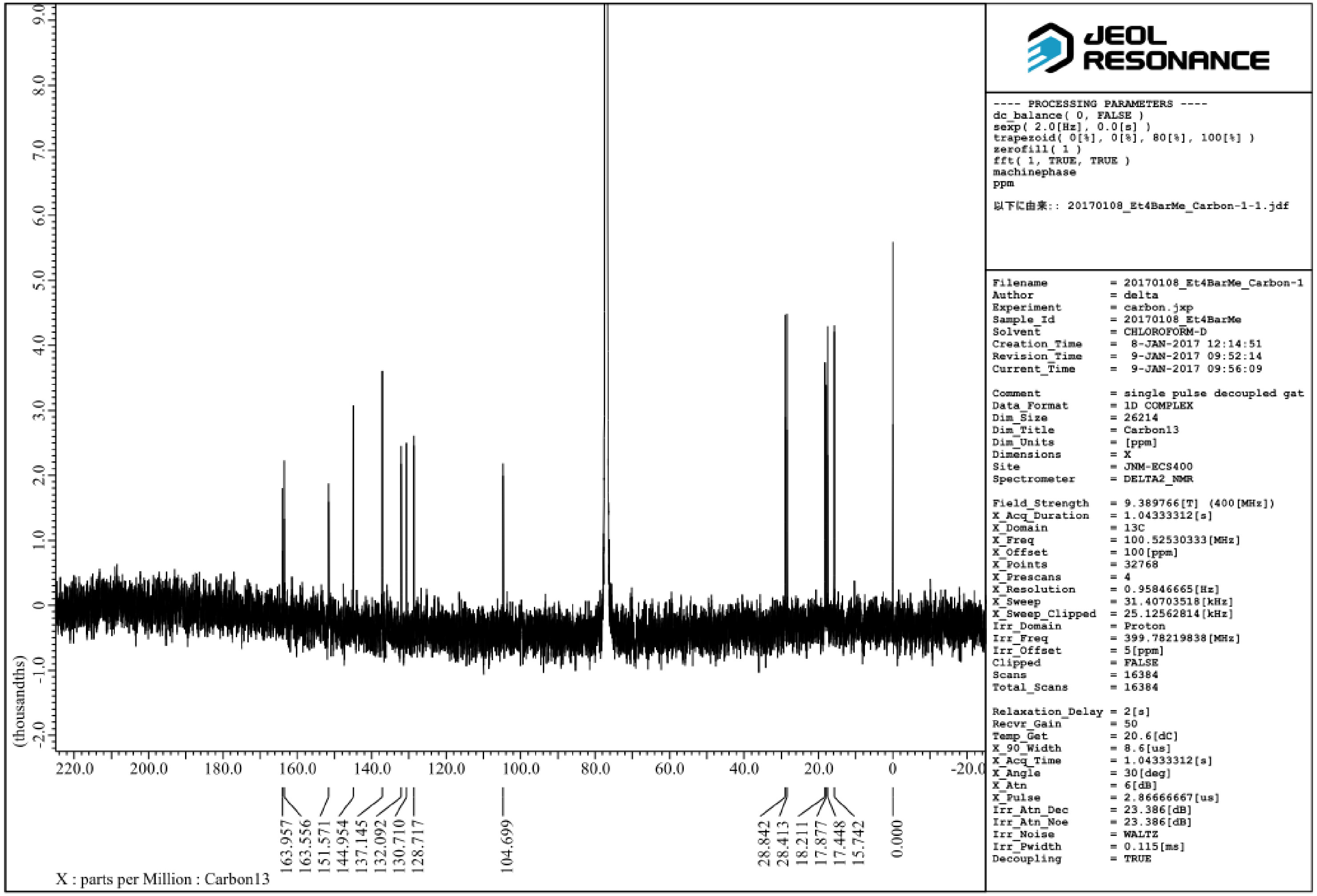

Figure S10. ^{13}C NMR spectrum of **4** in $CDCl_3$ at 25°C.

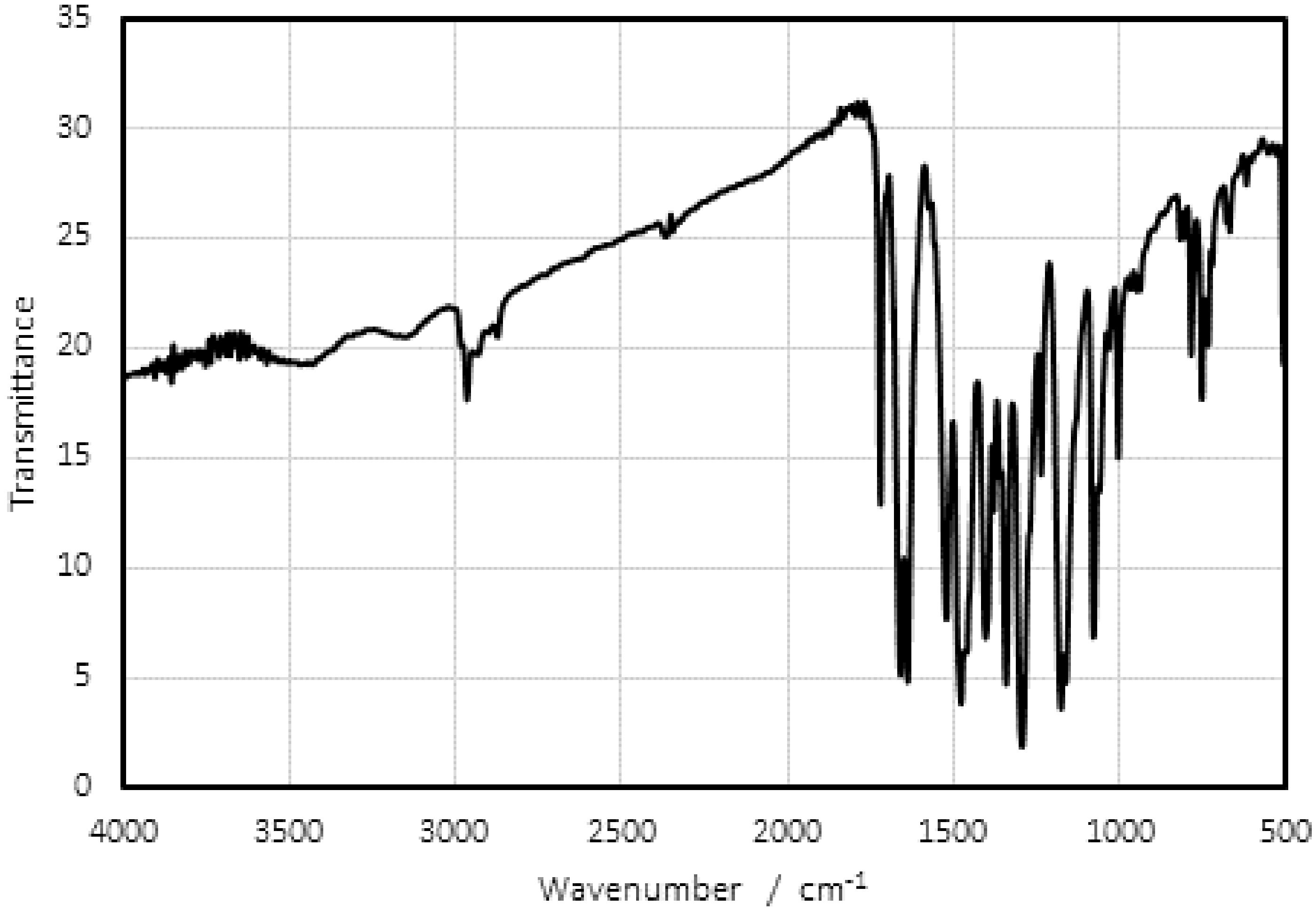

Figure S11. IR spectrum of **4**.

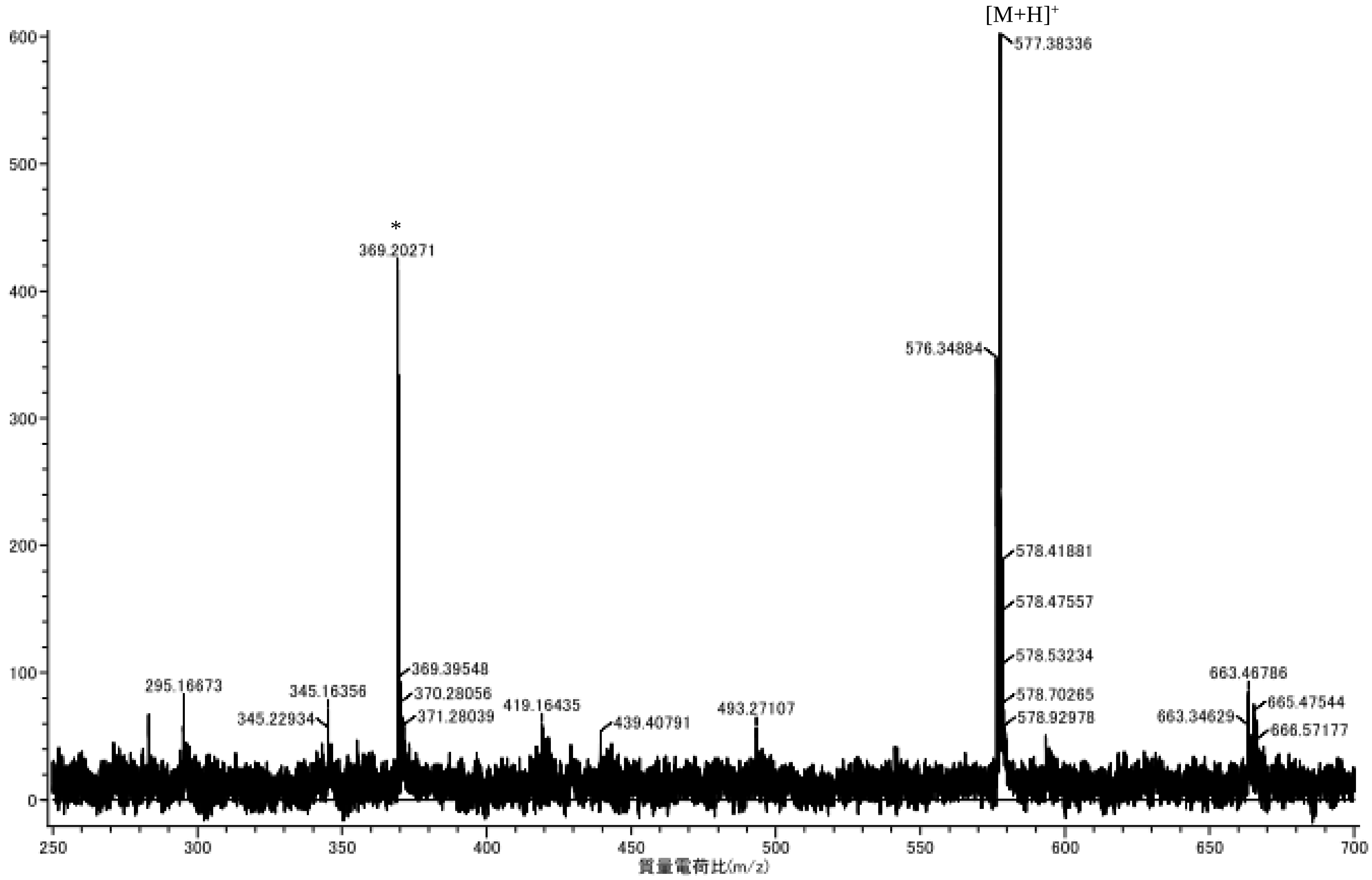

Figure S12. ESI-TOF-MS spectrum of **4** in CH_2Cl_2/CH_3OH. *Impurity in the solvents.

有機元素分析 CHN Corder MT-5/ﾔﾅｺ 利用者氏名 大河原 徹 No 206

試料名	秤取量(μg)	H (%)	C (%)	N (%)	残渣(μg)
CN4	1414.7	6.08	72.44	20.96	

備考: 分析日 2017/03/02

有機元素分析 CHN Corder MT-5/ﾔﾅｺ 利用者氏名 大河原 徹 No 203

試料名	秤取量(μg)	H (%)	C (%)	N (%)	残渣(μg)
MEL	1405.5	6.42	64.43	4.96	

備考: 分析日 2017/03/02

有機元素分析 CHN Corder MT-5/ﾔﾅｺ 利用者氏名 大河原 徹 No 39

試料名	秤取量(μg)	H (%)	C (%)	N (%)	残渣(μg)
BAL	1216.5	6.18	62.19	14.37	

備考: 分析日 2017/10/12

Figure S13. The elemental analyses of **2-4**.

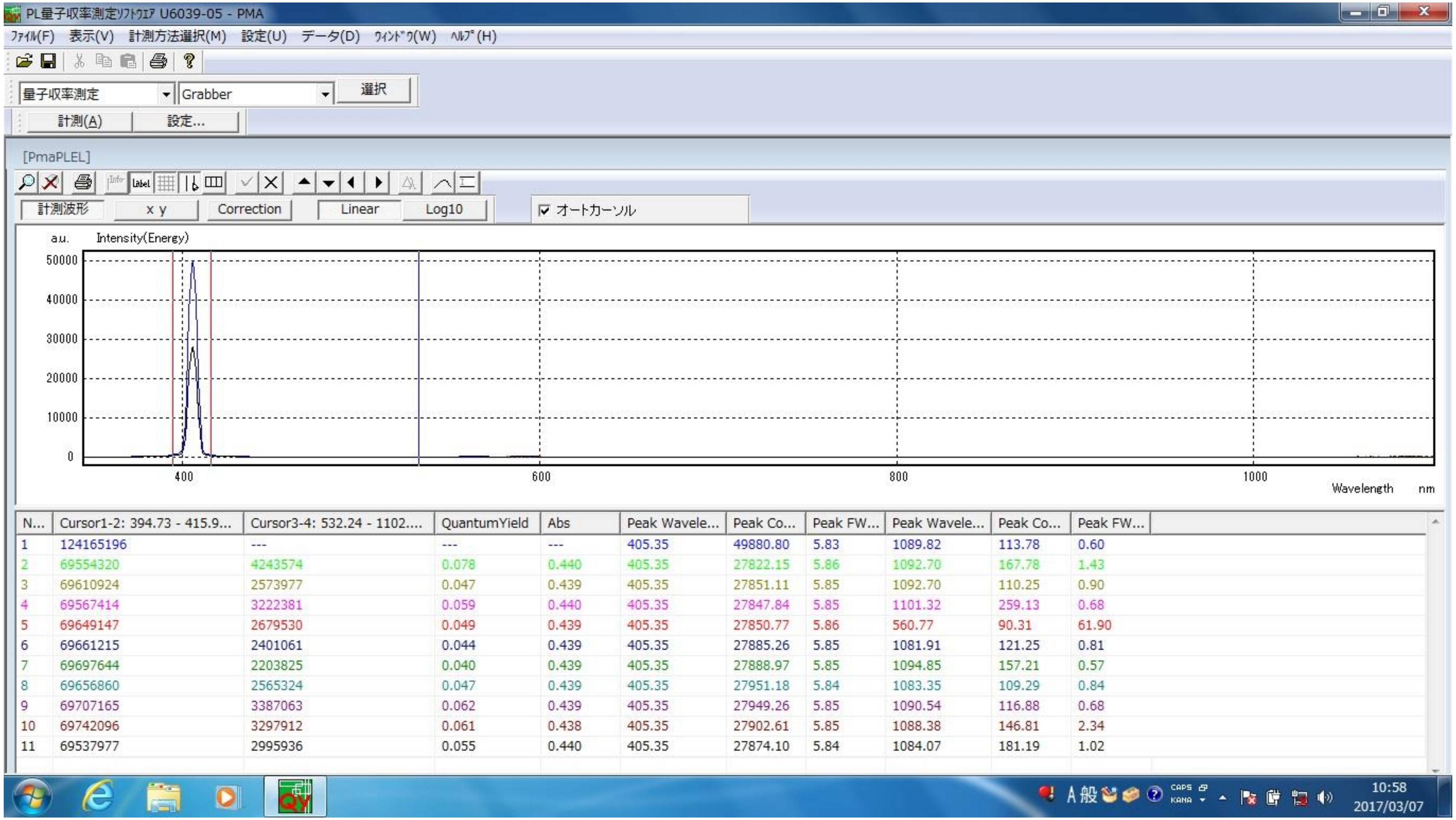

N...	Cursor1-2: 394.73 - 415.9...	Cursor3-4: 532.24 - 1102....	QuantumYield	Abs	Peak Wavele...	Peak Co...	Peak FW...	Peak Wavele...	Peak Co...	Peak FW...
1	124165196	---	---	---	405.35	49880.80	5.83	1089.82	113.78	0.60
2	69554320	4243574	0.078	0.440	405.35	27822.15	5.86	1092.70	167.78	1.43
3	69610924	2573977	0.047	0.439	405.35	27851.11	5.85	1092.70	110.25	0.90
4	69567414	3222381	0.059	0.440	405.35	27847.84	5.85	1101.32	259.13	0.68
5	69649147	2679530	0.049	0.439	405.35	27850.77	5.86	560.77	90.31	61.90
6	69661215	2401061	0.044	0.439	405.35	27885.26	5.85	1081.91	121.25	0.81
7	69697644	2203825	0.040	0.439	405.35	27888.97	5.85	1094.85	157.21	0.57
8	69656860	2565324	0.047	0.439	405.35	27951.18	5.84	1083.35	109.29	0.84
9	69707165	3387063	0.062	0.439	405.35	27949.26	5.85	1090.54	116.88	0.68
10	69742096	3297912	0.061	0.438	405.35	27902.61	5.85	1088.38	146.81	2.34
11	69537977	2995936	0.055	0.440	405.35	27874.10	5.84	1084.07	181.19	1.02

Figure S14. Summary of the quantum yield measurements of **2**.

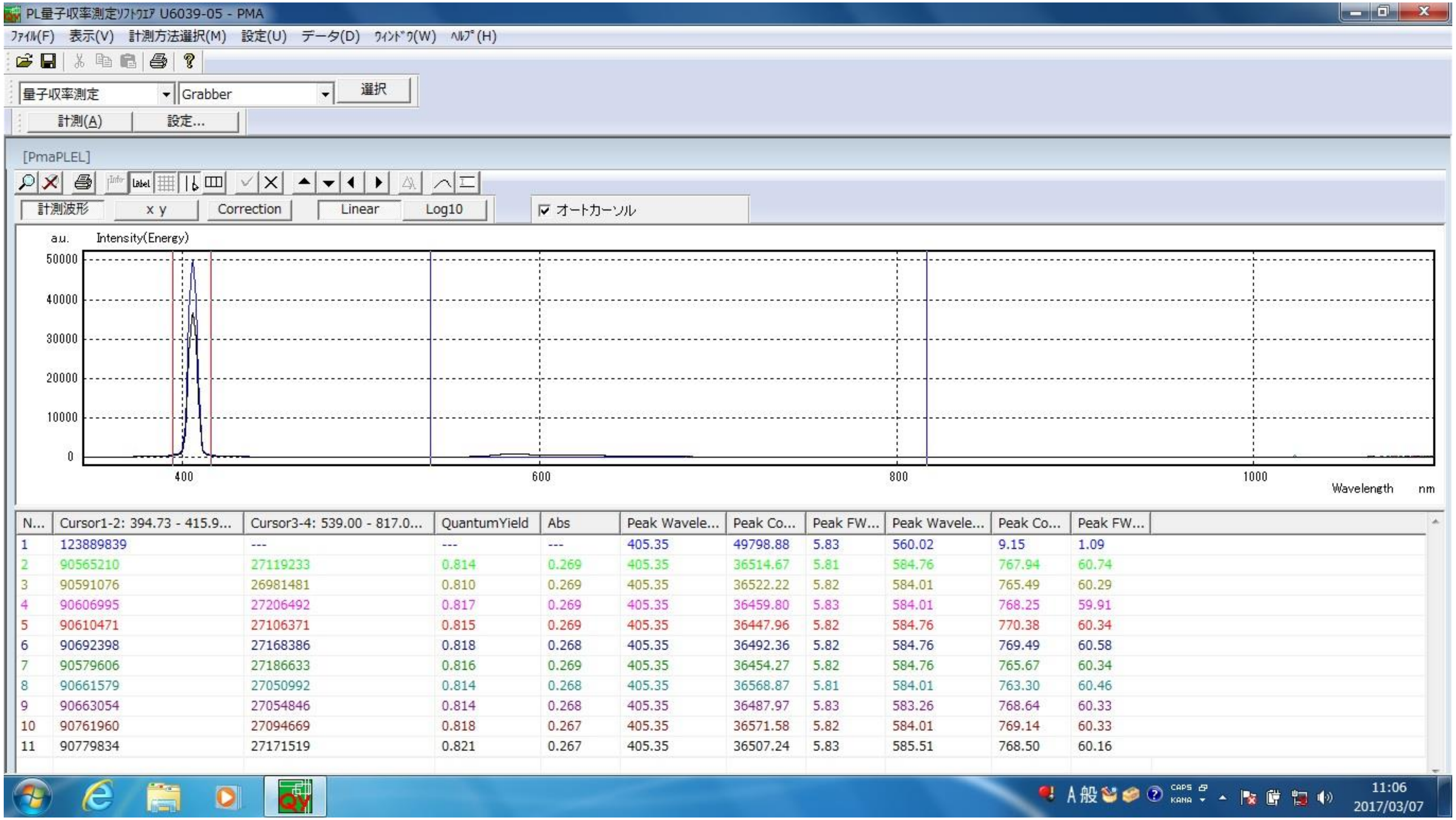

N...	Cursor1-2: 394.73 - 415.9...	Cursor3-4: 539.00 - 817.0...	QuantumYield	Abs	Peak Wavele...	Peak Co...	Peak FW...	Peak Wavele...	Peak Co...	Peak FW...
1	123889839	---	---	---	405.35	49798.88	5.83	560.02	9.15	1.09
2	90565210	27119233	0.814	0.269	405.35	36514.67	5.81	584.76	767.94	60.74
3	90591076	26981481	0.810	0.269	405.35	36522.22	5.82	584.01	765.49	60.29
4	90606995	27206492	0.817	0.269	405.35	36459.80	5.83	584.01	768.25	59.91
5	90610471	27106371	0.815	0.269	405.35	36447.96	5.82	584.76	770.38	60.34
6	90692398	27168386	0.818	0.268	405.35	36492.36	5.82	584.76	769.49	60.58
7	90579606	27186633	0.816	0.269	405.35	36454.27	5.82	584.76	765.67	60.34
8	90661579	27050992	0.814	0.268	405.35	36568.87	5.81	584.01	763.30	60.46
9	90663054	27054846	0.814	0.268	405.35	36487.97	5.83	583.26	768.64	60.33
10	90761960	27094669	0.818	0.267	405.35	36571.58	5.82	584.01	769.14	60.33
11	90779834	27171519	0.821	0.267	405.35	36507.24	5.83	585.51	768.50	60.16

Figure S15. Summary of the quantum yield measurements of **3**.

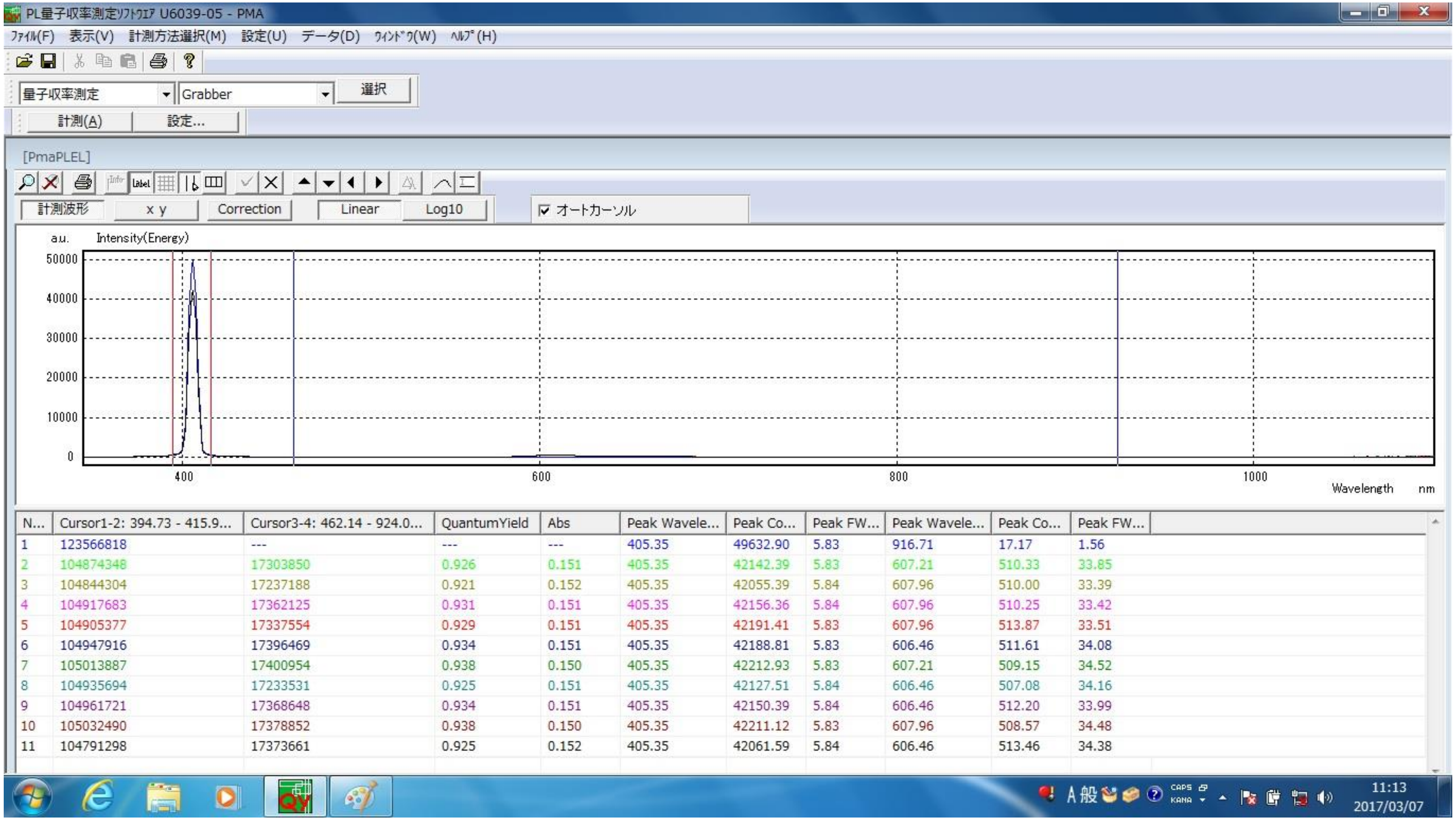

N...	Cursor1-2: 394.73 - 415.9...	Cursor3-4: 462.14 - 924.0...	QuantumYield	Abs	Peak Wavele...	Peak Co...	Peak FW...	Peak Wavele...	Peak Co...	Peak FW...
1	123566818	---	---	---	405.35	49632.90	5.83	916.71	17.17	1.56
2	104874348	17303850	0.926	0.151	405.35	42142.39	5.83	607.21	510.33	33.85
3	104844304	17237188	0.921	0.152	405.35	42055.39	5.84	607.96	510.00	33.39
4	104917683	17362125	0.931	0.151	405.35	42156.36	5.84	607.96	510.25	33.42
5	104905377	17337554	0.929	0.151	405.35	42191.41	5.83	607.96	513.87	33.51
6	104947916	17396469	0.934	0.151	405.35	42188.81	5.83	606.46	511.61	34.08
7	105013887	17400954	0.938	0.150	405.35	42212.93	5.83	607.21	509.15	34.52
8	104935694	17233531	0.925	0.151	405.35	42127.51	5.84	606.46	507.08	34.16
9	104961721	17368648	0.934	0.151	405.35	42150.39	5.84	606.46	512.20	33.99
10	105032490	17378852	0.938	0.150	405.35	42211.12	5.83	607.96	508.57	34.48
11	104791298	17373661	0.925	0.152	405.35	42061.59	5.84	606.46	513.46	34.38

Figure S16. Summary of the quantum yield measurements of **4**.

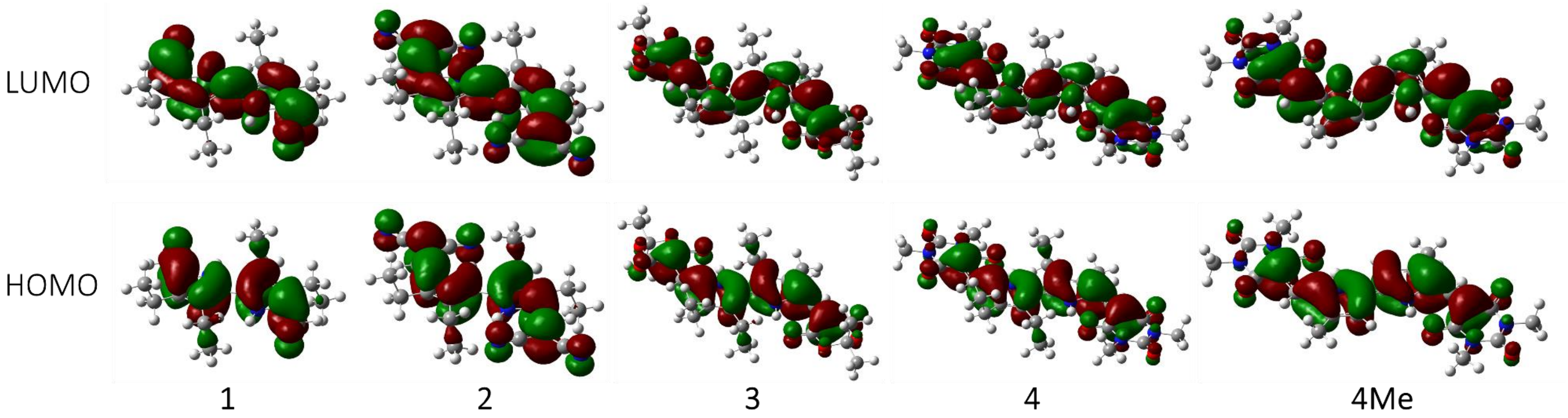

Figure S14. The HOMO and the LUMO orbitals of the model compounds **1-4** and **4Me**.

The initial coordinates of **1**

opt td rcam-b3lyp/6-31g(d)/auto scrf=(iefpcm,solvent=dichloromethane) geom=connectivity

Title Card Required

0 1

N	-1.64491568	-0.86910540	0.00035262
H	-1.50835226	-1.86767195	0.00507402
C	-0.68392454	0.12393503	0.00231344
C	-1.40905879	1.38318918	0.00293848
C	-2.76178127	1.09490922	0.00367695
C	-2.89779432	-0.31669102	0.00208736
C	-3.91489737	2.04970576	-0.00886880
H	-4.38482767	2.09545190	-0.99736559
H	-3.60395648	3.06181686	0.25640633
C	-4.04451616	-1.17751545	0.00331596
H	-5.03290592	-0.68695084	0.00315467
N	1.64380136	0.86868927	0.00424672
H	1.50652618	1.86716694	0.00377734
C	0.68332001	-0.12502537	0.00169481
C	1.40902240	-1.38420685	-0.00244760
C	2.76143924	-1.09539512	-0.00467480
C	2.89677451	0.31673884	-0.00011720
C	3.91786729	-2.04604841	-0.00371225
H	4.47165366	-1.99721875	0.94000644
H	3.59376508	-3.07853708	-0.14414402
C	4.04431032	1.17614071	0.00054374
H	5.03179139	0.68352126	-0.00177159
O	-3.93428673	-2.41137101	0.00305173
O	3.93600567	2.41031987	0.00434764
C	0.78618328	-2.74278200	-0.00205182
H	0.15713500	-2.90636780	-0.88516577
H	1.54969014	-3.52182800	-0.00761952
C	-0.78824572	2.74266273	-0.00133616
H	-0.15814543	2.90987233	0.88038485

H	-1.55360049	3.52000900	0.00267337
C	-0.08020722	-2.97741691	1.24931302
H	-1.04887024	-3.32204864	0.95296707
H	0.38476148	-3.71265680	1.87231094
H	-0.17848526	-2.06057073	1.79211548
C	0.07592687	2.97584500	-1.25450485
H	-0.38721948	3.71457108	-1.87472999
H	1.04698337	3.31529188	-0.96001359
H	0.16844808	2.05968819	-1.79947872
C	4.90951481	-1.71145091	-1.13343776
H	5.66270817	-1.04834592	-0.76210027
H	5.36856473	-2.61235063	-1.48351328
H	4.38627304	-1.24117619	-1.93963856
C	-5.00029064	1.61901396	0.99513619
H	-5.51732403	2.48417731	1.35439773
H	-5.69433227	0.96417282	0.51099851
H	-4.54335215	1.10944496	1.81759996

The initial coordinates of **2**

opt td rcam-b3lyp/6-31g(d)/auto scrf=(iefpcm,solvent=dichloromethane) geom=connectivity

Title Card Required

0 1

N	-1.84848814	-0.09859485	0.02569413
H	-2.08449619	-1.07584358	-0.05510246
C	-0.56731770	0.40386155	0.06982485
C	-0.68788825	1.84564408	0.11688071
C	-2.02871100	2.15314702	0.09121001
C	-2.76328660	0.92503114	0.02508713
C	-2.67522433	3.50021679	0.12744663
H	-3.29066558	3.66636620	-0.76269207
H	-1.93590140	4.30042383	0.16985013
C	-4.15657964	0.79804641	-0.03342859
H	-4.70835522	1.73070709	-0.02510424
N	1.84844280	0.09862012	0.02595583
H	2.08435931	1.07576602	-0.05636295
C	0.56726884	-0.40389786	0.06978716
C	0.68788168	-1.84568346	0.11683787
C	2.02870561	-2.15314252	0.09129313
C	2.76325969	-0.92499875	0.02514576
C	2.67537347	-3.50013056	0.12758463
H	3.32813906	-3.60214805	1.00058639
H	1.93618764	-4.30039402	0.17130637
C	4.15654013	-0.79804374	-0.03340736
H	4.70830741	-1.73071046	-0.02519736
C	-0.44763730	-2.81228317	0.20473024
H	-1.07559771	-2.79791276	-0.69333504
H	-0.08281886	-3.83370563	0.31572681
C	0.44761189	2.81225253	0.20486783
H	1.09147233	2.60761412	1.06688875
H	0.08276508	3.83357741	0.31665565
C	4.92202911	0.36635913	-0.10422859

C	-4.92204040	-0.36636025	-0.10426671
C	6.33856992	0.28444108	-0.15873046
C	4.36157950	1.66705109	-0.12898407
C	-4.36154637	-1.66703291	-0.12910630
C	-6.33859087	-0.28447964	-0.15864074
N	7.49644577	0.21041697	-0.20295923
N	3.86628541	2.71947960	-0.14822230
N	-3.86625780	-2.71946371	-0.14832545
N	-7.49647160	-0.21048178	-0.20275685
C	-3.59208778	3.64452956	1.35632224
H	-3.45171551	4.61051382	1.79458019
H	-4.61278385	3.53464455	1.05465221
H	-3.34880516	2.88869949	2.07356312
C	1.32953198	2.79287589	-1.05744810
H	0.71994166	2.95887051	-1.92101364
H	2.06795260	3.56416251	-0.98851381
H	1.81346408	1.84213161	-1.13988015
C	-1.35244500	-2.52523429	1.41738269
H	-2.20377874	-1.95941910	1.10118017
H	-1.67800468	-3.44987003	1.84628787
H	-0.80421637	-1.96697076	2.14723692
C	3.53970718	-3.73442349	-1.12526575
H	4.57103163	-3.59073967	-0.87904464
H	3.39310706	-4.73395299	-1.47789757
H	3.25552612	-3.04136567	-1.88934164

The initial coordinates of **3**

opt td cam-b3lyp/6-31g(d)/auto scrf=(iefpcm,solvent=dichloromethane) geom=connectivity

kct43

0 1

N	1.83315674	0.23569607	0.09525473
H	2.31102462	-0.65203760	0.25245488
C	0.48969803	0.49668187	-0.00808640
C	0.35132935	1.93610198	-0.12960622
C	1.61293491	2.47843766	-0.08274915
C	2.55078827	1.39893530	0.06138489
C	2.01036459	3.91675235	-0.17096329
H	1.14295444	4.56846856	-0.28134550
H	2.66906234	4.09327769	-1.02751799
C	3.94248827	1.54531565	0.14499736
H	4.27191193	2.57871625	0.12989342
C	5.00338541	0.64217147	0.23164474
C	4.90274130	-0.80284260	0.31713657
C	6.33486033	1.25327091	0.34476661
C	7.24143605	-0.88785860	-0.23600365
C	7.16593637	-0.81409729	-1.75269279
H	7.06238194	-1.81956304	-2.16605488
H	8.07980331	-0.36147938	-2.14317445
H	6.31222631	-0.21253397	-2.07496020
C	8.40284300	-1.71310568	0.26716170
H	8.39773602	-1.72622614	1.35883617
H	9.34222173	-1.28050706	-0.08255895
H	8.32033887	-2.73596678	-0.10549878
O	3.87612200	-1.45551039	0.46860377
O	6.54109499	2.43813297	0.50824231
O	6.06055173	-1.50218431	0.28462830
O	7.40282691	0.41481529	0.31590405
N	-1.83315327	-0.23568858	-0.09525662
H	-2.31102206	0.65204540	-0.25245218

C	-0.48969426	-0.49667371	0.00808330
C	-0.35132455	-1.93609407	0.12959986
C	-1.61292979	-2.47843048	0.08274115
C	-2.55078410	-1.39892834	-0.06138950
C	-2.01035836	-3.91674578	0.17095069
H	-1.14294763	-4.56846169	0.28133017
H	-2.66905536	-4.09327439	1.02750530
C	-3.94248399	-1.54531066	-0.14500160
H	-4.27190497	-2.57871216	-0.12990108
C	-5.00338454	-0.64216985	-0.23164454
C	-4.90274763	0.80284512	-0.31713122
C	-6.33485678	-1.25327520	-0.34476723
C	-7.24144219	0.88784807	0.23601191
C	-7.16594018	0.81408200	1.75270071
H	-7.06239046	1.81954688	2.16606607
H	-8.07980432	0.36145800	2.14318193
H	-6.31222657	0.21252201	2.07496499
C	-8.40285374	1.71309127	-0.26714920
H	-8.39774851	1.72621501	-1.35882364
H	-9.34222994	1.28048724	0.08257164
H	-8.32035379	2.73595163	0.10551423
O	-3.87613227	1.45551896	-0.46859870
O	-6.54108600	-2.43813777	-0.50824553
O	-6.06056146	1.50218100	-0.28461957
O	-7.40282749	-0.41482475	-0.31589992
C	0.93473037	-2.67865993	0.28449293
H	1.61277278	-2.50698675	-0.55756994
H	0.75781027	-3.75310455	0.34316287
C	-0.93472485	2.67866828	-0.28450266
H	-1.61276647	2.50700182	0.55756222
H	-0.75780356	3.75311230	-0.34318021
C	-2.77097641	-4.36319917	-1.09148329
H	-2.33026791	-5.25959269	-1.47509075
H	-3.79564192	-4.54705849	-0.84418519
H	-2.71550055	-3.59314431	-1.83231926
C	1.68529981	-2.26892561	1.56525961

H	2.63371660	-2.76332513	1.59647995
H	1.83512291	-1.20946889	1.56738159
H	1.10855921	-2.54929741	2.42180029
C	-1.68529642	2.26892604	-1.56526557
H	-1.83879881	1.20999403	-1.56539243
H	-2.63195384	2.76655223	-1.59855081
H	-1.10663739	2.54554097	-2.42173384
C	2.77098204	4.36320946	1.09146974
H	3.79748507	4.53898755	0.84592222
H	2.33553079	5.26419999	1.47028141
H	2.70841096	3.59664140	1.83535029

The initial coordinates of **4**

opt td cam-b3lyp/6-31g(d)/auto scrf=(iefpcm,solvent=dichloromethane) geom=connectivity

kct43

0 1

N	-1.83568836	-0.22854887	0.00023297
H	-2.32971978	0.66661526	0.00007147
C	-0.49058968	-0.49603945	0.00029141
C	-0.35096572	-1.94023903	0.00036826
C	-1.61534940	-2.47745437	0.00034350
C	-2.55560739	-1.38917808	0.00023215
C	-2.01415079	-3.91809190	0.00044306
H	-1.14517808	-4.57709499	0.00009671
H	-2.61608191	-4.16213473	-0.88104480
C	-3.94988339	-1.53139425	0.00016070
H	-4.28004837	-2.56467946	0.00025281
C	-5.02017709	-0.63480257	-0.00000607
C	-4.93312331	0.80830266	-0.00021126
C	-6.34298918	-1.26364979	0.00002857
C	-7.39636089	0.95966141	-0.00034175
O	-3.88077036	1.46394579	-0.00029132
O	-6.51963040	-2.47808913	0.00027135
N	1.83572243	0.22864256	0.00022399
H	2.32974157	-0.66652836	0.00005311
C	0.49062782	0.49614982	0.00032465
C	0.35102287	1.94035253	0.00050829
C	1.61541429	2.47755106	0.00050759
C	2.55565651	1.38926163	0.00029408
C	2.01423515	3.91818312	0.00065410
H	1.14527124	4.57719758	0.00133126
H	2.61635609	4.16201771	0.88206936
C	3.94993464	1.53144512	0.00018604
H	4.28013310	2.56471964	0.00031093
C	5.02019009	0.63480973	-0.00005846

C	4.93305899	-0.80829015	-0.00029538
C	6.34303390	1.26358978	-0.00002787
C	7.39628840	-0.95977712	-0.00048900
O	3.88066818	-1.46387286	-0.00033638
O	6.51973824	2.47802002	0.00022611
O	8.40274071	-1.64872240	-0.00059006
O	-8.40284904	1.64855428	-0.00045218
N	-7.45143449	-0.42276375	-0.00025188
N	-6.12565233	1.52097520	-0.00031025
N	6.12555067	-1.52102538	-0.00051492
N	7.45143475	0.42264535	-0.00038997
C	6.01855622	-2.97921058	-0.00070477
H	5.47546664	-3.31004933	0.88568078
H	5.47489242	-3.30974506	-0.88684589
H	7.02458239	-3.38665242	-0.00108976
C	8.76288578	1.06692153	-0.00040321
H	8.86467308	1.69598805	-0.88590441
H	8.86579631	1.69401532	0.88638857
H	9.51897265	0.28789121	-0.00169930
C	-6.01873397	2.97916598	-0.00044732
H	-5.47521639	3.30994174	0.88569415
H	-5.47553273	3.30982022	-0.88683267
H	-7.02478141	3.38655534	-0.00030501
C	-8.76285160	-1.06710915	-0.00026864
H	-8.86473674	-1.69594452	-0.88592553
H	-8.86559761	-1.69444513	0.88636812
H	-9.51897963	-0.28811838	-0.00125713
C	-0.93927450	2.69214049	0.00072681
H	-1.54766814	2.46363077	-0.88012044
H	-0.76100265	3.76805155	0.00103876
C	0.93934480	-2.69200517	0.00051396
H	1.54770767	-2.46307233	0.88127390
H	0.76109245	-3.76791942	0.00069046
C	-2.85983930	-4.26141875	1.24082107
H	-3.86605989	-4.46714429	0.94065926
H	-2.44907025	-5.12244905	1.72537821

H	-2.85197626	-3.43320805	1.91824650
C	1.79528164	-2.37035085	-1.23864327
H	1.98919343	-1.31869586	-1.27497486
H	2.72194902	-2.90207377	-1.17983589
H	1.26941155	-2.66679595	-2.12209160
C	-1.79517873	2.36992353	1.23976038
H	-2.71835947	2.90813573	1.18530904
H	-1.99607780	1.31942001	1.27109589
H	-1.26578532	2.65833696	2.12376282
C	2.85966516	4.26178055	-1.23982521
H	2.45393261	5.12809902	-1.71917205
H	3.86794874	4.45891842	-0.94082539
H	2.84452303	3.43705734	-1.92136930

The initial coordinates of **4Me**

# opt td cam-b3lyp/6-31g(d)/auto scrf=(iefpcm,solvent=dichloromethane)
geom=connectivity

kct43

0 1

N	-1.83251812	-0.22204942	0.00000210
H	-2.32150643	0.67905352	-0.00004566
C	-0.49107095	-0.49224312	-0.00011235
C	-0.35317140	-1.91754606	-0.00030406
H	0.58803865	-2.45061458	-0.00041070
C	-1.60222838	-2.47379631	-0.00037504
C	-2.55111264	-1.38359592	-0.00021489
C	-1.95640622	-3.92397348	-0.00064935
H	-1.05476698	-4.53959054	0.00004527
H	-2.54814036	-4.18977327	-0.88279944
H	-2.54952130	-4.18978730	0.88056089
C	-3.94563965	-1.51870174	-0.00028861
H	-4.28570834	-2.54898882	-0.00047637
C	-5.00354008	-0.60726408	-0.00014699
C	-4.90162778	0.83732025	0.00007641
C	-6.33367556	-1.22236582	-0.00028023
C	-7.36493634	1.01018143	0.00030483
O	-3.84618676	1.48765377	-0.00007931
O	-6.52121920	-2.43507478	-0.00040261
N	1.83252810	0.22206768	-0.00001176
H	2.32151550	-0.67903525	0.00079223
C	0.49108098	0.49226337	-0.00004460
C	0.35318394	1.91756662	-0.00014893
H	-0.58802527	2.45063665	-0.00030196
C	1.60224178	2.47381506	-0.00014221
C	2.55112409	1.38361327	-0.00000560
C	1.95642156	3.92399183	-0.00024652
H	1.05478331	4.53960972	-0.00134493
H	2.54795244	4.19007278	0.88195560

H	2.54974058	4.18952306	-0.88140419
C	3.94565116	1.51871432	0.00004813
H	4.28572546	2.54899956	-0.00006734
C	5.00354484	0.60726900	0.00004838
C	4.90161598	-0.83731327	0.00063127
C	6.33368741	1.22235727	-0.00033924
C	7.36492120	-1.01020270	0.00005140
O	3.84616601	-1.48763254	0.00132618
O	6.52124508	2.43506390	-0.00050607
O	8.36441272	-1.70886674	0.00019120
O	-8.36443754	1.70883152	0.00060538
N	-7.43368398	-0.37149845	-0.00031486
N	-6.08864051	1.55925957	0.00054266
N	6.08861992	-1.55926644	0.00053332
N	7.43368525	0.37147776	-0.00058278
C	5.96889436	-3.01664483	0.00126122
H	5.42341962	-3.34205858	0.88817331
H	5.42185045	-3.34274403	-0.88441908
H	6.97128916	-3.43285923	0.00055881
C	8.75134342	1.00314644	-0.00109150
H	8.85858680	1.63135325	-0.88653405
H	8.86070415	1.62886658	0.88589330
H	9.49985252	0.21683818	-0.00298934
C	-5.96892992	3.01663949	0.00104168
H	-5.42177880	3.34197492	0.88693408
H	-5.42356916	3.34282925	-0.88565802
H	-6.97132869	3.43284306	0.00222105
C	-8.75133506	-1.00318208	-0.00057515
H	-8.85900549	-1.63090718	-0.88631592
H	-8.86025186	-1.62939053	0.88611124
H	-9.49985348	-0.21688097	-0.00172622
